# Synthesis of Purine-Based Ionic Liquids and Their Applications

**DOI:** 10.3390/molecules26226958

**Published:** 2021-11-18

**Authors:** Ana R. F. Carreira, Telma Veloso, Nicolas Schaeffer, Joana L. Pereira, Sónia P. M. Ventura, Cécile Rizzi, Juliette Sirieix Plénet, Helena Passos, João A. P. Coutinho

**Affiliations:** 1 Department of Chemistry, CICECO-Aveiro Institute of Materials, University of Aveiro, 3810-193 Aveiro, Portugal; ritafutre@ua.pt (A.R.F.C.); telmaveloso@ua.pt (T.V.); nicolas.schaeffer@ua.pt (N.S.); spventura@ua.pt (S.P.M.V.); jcoutinho@ua.pt (J.A.P.C.); 2 Department of Biology & CESAM, University of Aveiro, 3810-193 Aveiro, Portugal; jpereira@ua.pt; 3 Laboratoire Physico-Chimie des Électrolytes et Nano-Systèmes Interfaciaux, PHENIX, CNRS, Sorbonne Université, F-75005 Paris, France; cecile.rizzi@upmc.fr (C.R.); juliette.sirieix_plenet@sorbonne-universite.fr (J.S.P.)

**Keywords:** synthesis, ecotoxicity, solubility, liquid–liquid equilibrium, thermoresponsive systems

## Abstract

Bio-based ionic liquids (ILs) are being increasingly sought after, as they are more sustainable and eco-friendly. Purines are the most widely distributed, naturally occurring *N*-heterocycles, but their low water-solubility limits their application. In this work, four purines (theobromine, theophylline, xanthine, and uric acid) were combined with the cation tetrabutylammonium to synthesize bio-based ILs. The physico–chemical properties of the purine-based ILs were characterized, including their melting and decomposition temperatures and water-solubility. The ecotoxicity against the microalgae *Raphidocelis subcapitata* was also determined. The ILs show good thermal stability (>457 K) and an aqueous solubility enhancement ranging from 53- to 870-fold, in comparison to their respective purine percursors, unlocking new prospects for their application where aqueous solutions are demanded. The ecotoxicity of these ILs seems to be dominated by the cation, and it is similar to chloride-based IL, emphasizing that the use of natural anions does not necessarily translate to more benign ILs. The application of the novel ILs in the formation of aqueous biphasic systems (ABS), and as solubility enhancers, was also evaluated. The ILs were able to form ABS with sodium sulfate and tripotassium citrate salts. The development of thermoresponsive ABS, using sodium sulfate as a salting-out agent, was accomplished, with the ILs having different thermosensitivities. In addition, the purine-based ILs acted as solubility enhancers of ferulic acid in aqueous solution.

## 1. Introduction

Ionic liquids (ILs) are alternative solvents, composed of a large organic cation (e.g., tetraalkylammonium, tetraalkylphosphonium, imidazolium, cholinium, pyridinium) and an organic or inorganic anion [1]. Cation–anion asymmetry and charge dispersion, in at least one of the ions, reduces the electrostatic interactions and crystallization ability, creating salts with low melting points. In fact, the first definition of ILs was proposed by Walden et al. [2] as salts with melting temperatures below an arbitrary threshold of 373 K, in order to differentiate these from typical salts. More recently, the definition of ILs was refined by Mariani et al. [3], who proposed that ILs are water-free organic salts with a melting temperature lower than decomposition temperature.

Irrespective of their definition, the ionic character of ILs enables different cation–anion arrangements and a consequent formulation of unique, tunable, and versatile compounds. The structural diversity of ILs resulted in the upsurge of a great number of applications, such as metal recovery, biomolecules extraction, protein stabilization, CO_2_ capture, batteries, chromatography, catalysis, hydrotropy, and separation processes (e.g., by the formation of aqueous biphasic systems (ABS)), among others [4,5,6]. Although ILs have been considered attractive non-volatile, non-flammable, and thermally and chemically stable compounds, their sustainability and biocompatibility has been questioned [7,8]. They often do not fulfill more than just a few of the twelve principles of green chemistry [7,9]. Aiming to fulfill the 7th and 10th principles of green chemistry [9] (namely the use of renewable feedstock and design for degradation, respectively), the use of natural and renewable building blocks for the design of ILs is being increasingly targeted [10,11,12,13,14]. Among these, organic acids [10], natural sugars [11], amino acids [12], terpenes [13], and purines [14] are some examples of the natural building blocks used for their synthesis.

Purines are *N*-heterocycles comprising of two rings. They are widely distributed in nature [15], including in tea, cocoa beans, coffee [16], and human and animal tissues [17]. Despite their widespread natural occurrence, purines have, in general, low solubility in both water and organic solvents, which limits their application [18,19]. This issue can be overcome by forming salts of purines [20,21,22]. Salt formation is often considered advantageous, due to its good stability, solubility, easy formulation, and facilitated crystallization, increasing the potential application of these compounds [22]. By combining purines with different salts (e.g., sodium, lithium, phosphate, and mesylate, among others) it is possible to improve their water-solubility [20,21,22]. This unlocks new prospects for the application of purines, including their use as building blocks for the synthesis of ILs. By using purines as feedstock, it may be possible to design novel, bio-based ILs, while simultaneously increasing the purine solubility. Gavhane et al. [14] were able to accomplish this through the synthesis of water-soluble ILs, based on purines, by using 1-*n*-alkyl-3-methylimidazolium as a cation and adenine, guanine, hypoxanthine, or xanthine as anions. As the design of ILs based on purines seems to eliminate the solubility obstacles for the use of purines in aqueous solution, it becomes possible to conceive of novel applications for purine-based salts.

Aqueous solutions of ILs can be used to induce the formation of ABS and enhance the solubility of poorly water-soluble molecules. ABS are composed of two aqueous immiscible phases, based on polymer–polymer, salt–polymer, or salt–salt combinations [23]. These are primarily composed of water and are considered to be a sustainable alternative to conventional liquid–liquid extraction systems [24]. ILs can be used to form ABS for the most diverse applications, such as chiral resolution [25], metal separation [26], capillary electrophoresis [27], and the extraction and separation of proteins [28,29]. ABS can be customized to enhance the selective extraction and separation of (bio)molecules and parameters, as phase forming component concentration, pH, and temperature can be adjusted to maximize their efficiency [30,31]. Recently, aqueous solutions of ILs have also emerged as potential (bio)molecule solubility enhancers [32]. The application of aqueous solutions of ILs as solubility enhancers can be used for the extraction and recovery of bioactive molecules from biomass [33], improvement of the bioavailability of pharmaceuticals [4], and drug formulation [34].

In this work, three novel purine-based ILs and one salt were synthesized, using tetrabutylammonium as a cation and theophylline, theobromine, xanthine, and uric acid as anions (Table 1). They were further characterized, regarding melting and decomposition temperatures, water-solubility, and ecotoxicity against the microalgae *Raphidocelis subcapitata*. Aiming to expand the application of the synthesized bio-based ILs, their ability to promote ABS formation in the presence of two different salts (sodium sulfate (Na_2_SO_4_) and tripotassium citrate (K_3_C_6_H_5_O_7_)) at different temperatures was determined. Finally, the capacity of these compounds to enhance the solubility of 4-hydroxy-3-methoxycinnamic acid, also known as ferulic acid, in aqueous solution was also studied.

## 2. Experimental Section

### 2.1. Materials

Four bio-based salts were synthesized, using purines as anions, namely tetrabutylammonium theobrominate [N_4444_][Theob], tetrabutylammonium theophyllinate [N_4444_][Theop], tetrabutylammonium xanthinate [N_4444_][Xan], and tetrabutylammonium urate [N_4444_][Ur]. Tetrabutylammonium hydroxide ([N_4444_]OH, in aqueous solution at 40 wt%), tetrabutylammonium chloride (97 wt% pure), theobromine (99 wt% pure), and xanthine (99 wt% pure) were acquired from Sigma Aldrich (Saint Louis, MO, USA). Theophylline (99 wt% pure) and tripotassium citrate monohydrate (K_3_C_6_H_5_O_7_, 99 wt% pure) were acquired from Acros Organic (Waltham, MA, USA); uric acid (99 wt% pure) from Alfa Aesar (Haverhill, MA, USA); and sodium sulfate anhydrous (Na_2_SO_4_, 99 wt% pure) from José Manuel Gomes dos Santos (Odivelas, Portugal). The 4-hydroxy-3-methoxycinnamic acid (hereinafter referred to as ferulic acid, 99 wt% pure) was purchased from TCI. The used water was double distilled, passed through a reverse osmosis system, and further treated with a Milli-Q plus 185 water purification apparatus.

### 2.2. Synthesis and Characterization of Purine-Based ILs

Four purines, namely theobromine, theophylline, xanthine, and uric acid, were converted into salts through a well-established neutralization protocol [35]. [N_4444_]OH (1 equivalent, 40 wt% in aqueous solution) was slowly added to an aqueous solution of the purine, with a molar excess of 1.1 equivalents. The reaction mixture was stirred for 2 h at room temperature. As a result, the respective salt was obtained, and water was formed as a byproduct. The water was removed under reduced pressure. The resultant product was dissolved in acetonitrile and filtered to remove the excess purine. Lastly, acetonitrile was removed under reduced pressure, and the obtained compound was dried under high vacuum for at least 48 h. All the salts were obtained as white solids, except for [N_4444_][Xan], which was obtained as a pale beige solid. The same protocol was used, in the attempt to produce fully bio-based ILs by using cholinium cation, instead of tetrabutylammonium and theophylline as the anion; however, this was not successful. The structure of the synthesized salts was confirmed by ^1^H and ^13^C nuclear magnetic resonance (NMR) spectroscopy, as reported in the Supplementary Materials. The NMR spectra were recorded with 300.13 MHz (^1^H) and 75.47 MHz (^13^C) on a Bruker Avance III NMR spectrometer (Spectrum S1 to S8). Tetramethylsilane was added as an internal reference, and D_2_O was used as a solvent.

### 2.3. Melting and Degradation Temperatures

The melting temperatures (T_m_) were measured in a differential scanning calorimetry (DSC), using a Hitachi DSC7000X equipment at atmospheric pressure, with hermetically sealed aluminum crucibles and a constant flow of nitrogen. The equipment was previously calibrated using compounds with mass fractions purities higher than 99%. Each sample underwent three cycles of cooling and heating, at 5 K∙min^−1^ and 2 K∙min^−1^, respectively. The melting point of each compound was taken as the peak temperature. The determined melting points are estimated to have a 1 K uncertainty. When necessary, melting points were also measured in a Bücchi device, model M-565, with a temperature resolution of 0.1 K. The degradation temperature (T_d_) of the purine-based salts was measured using a Setsys Evolution 1750 (SETARAM) instrument. Each sample was heated in an aluminia pan, under a nitrogen atmosphere, with a heating rate of 2 K·min^−1^. The degradation temperature-associated uncertainty is estimated to be 0.01 K. Degradation temperature was taken as the peak temperature of the derivative.

### 2.4. Solubility Assays

IL solubility studies were performed using the isothermal shake-flask method, as reported in the literature [36]. The ILs [N_4444_][Theob], [N_4444_][Theop], [N_4444_][Xan], or the salt [N_4444_][Ur] were added in excess of 0.5 mL of water, forming a saturated aqueous solution. The samples were stirred at 1050 rpm at (298 ± 1) K for 72 h using the Eppendorf Thermomixer comfort equipment. After that time, equilibrium was reached, the stirring was turned off, and samples were left undisturbed at 298 K, in order to enable the separation of the excess undissolved solute and the liquid phase. If needed, samples were centrifuged for 2 min at 12,000 rpm. Samples of the liquid phase were collected and diluted in distilled water, followed by the preparation of a calibration curve for each compound of interest. The amount of compound in the liquid phase was quantified using a UV–Vis Synergy HT microplate reader from BioTek (Winooski, VT, USA). Both [N_4444_][Theob] and [N_4444_][Theop] were quantified at 272 nm, while [N_4444_][Xan] and [N_4444_][Ur] were quantified at 267 nm and 290 nm, respectively. All assays were performed in triplicate. The pH (±0.02) of the samples was determined by using a Mettler Toledo SevenMultiTMdual pH meter.

The ferulic acid solubility studies were performed by adding it in excess to aqueous solutions of [N_4444_][Theob], [N_4444_][Theop], or [N_4444_]Cl, with the following concentrations: 0.05, 0.1, 0.25, 0.5, 1.0, and 1.5 mol L^−1^. The samples were stirred at 1150 rpm at (303 ± 1) K for 72 h. After that time, equilibrium was reached, the stirring was turned off, and samples were left undisturbed at 303 K. If needed, samples were centrifuged for 2 min at 12,000 rpm. Samples were quantified, as previously described, at 316 nm (see Figure S1). All assays were performed in triplicates. To verify the integrity of the IL, the liquid phase was analyzed by ^1^H and ^13^C NMR.

The effect of pH on the water-solubility of ferulic acid was conducted by adjusting the initial pH of the aqueous solutions with NaOH, followed by the addition of ferulic acid until saturation was completed. The samples were stirred at 1150 rpm at (303 ± 1) K for 72 h, and pH was remeasured. When necessary, NaOH was re-added to the solutions, aiming to achieve a wide range of pH values. The assays were conducted in duplicates.

### 2.5. Microalgae Ecotoxicity Assays

The microalgae ecotoxicity assays were conducted, as previously described in detail [37], using the microalgae *Raphidocelis subcapitata*, i.e., following the guidelines of OECD [38], and adapted to the use of 24-well microplates [39]. Briefly, the initial test cell density of 10^4^ cells·mL^−1^ was obtained by microscopic cell counting (Neubauer hemocytometer) of a microalgae inoculum culture, grown in MBL medium [40], under controlled incubation conditions (296 ± 1) K and permanent illumination of 7000 lux. The microalgae were exposed to a range of concentrations of each salt, and all the assays, including the MBL blank control, were conducted in triplicate. The microplates were incubated for 96 h, under controlled incubation conditions, as used for the inoculum. After that time, the microalgae yield in each treatment was calculated as the difference between the cell densities at the end and beginning of the test. EC_50_, and the corresponding 95% confidence intervals, were calculated through a non-linear regression, using the least squares method to fit the data to the logistic equation.

### 2.6. Aqueous Biphasic Systems

The ABS ternary phase diagrams, composed of [N_4444_][Theob] or [N_4444_][Theop], Na_2_SO_4_ or K_3_C_6_H_5_O_7_, and water were determined through the cloud point titration method [41,42] at atmospheric pressure, continuous stirring, and in a temperature-controlled cell at (298, 323 or 353) K ± 1 K for the Na_2_SO_4_ salt and at (298 ± 1) K for the K_3_C_6_H_5_O_7_ salt. [N_4444_]Cl ternary phase diagrams, using Na_2_SO_4_ as a salting-out agent, were assessed under the same conditions described above, as a means of comparison. Each IL solution was prepared at 60 wt%, and the salting-out agents Na_2_SO_4_ and K_3_C_6_H_5_O_7_ solutions were prepared at 17 and 40 wt%, respectively. Briefly, the salt aqueous solution was added dropwise to a known amount of IL aqueous solution, until the mixture became turbid. Then, water was added to the cloudy mixture until it became clear, fitting the monophasic region. The inverse methodology was also used, when necessary, by adding an aqueous solution of the IL dropwise to a known aqueous solution of salt, to further complete the profile of the binodal curve. The ternary systems composition was determined by weight quantification (±10^−4^ g) of all the added components. Tie-lines (TL) and tie-line lengths (TLL) were determined by applying the gravimetric method (±10^−4^ g), proposed by Merchuk et al. [43]. Biphasic mixtures, composed of IL, salt, and water, were prepared and vigorously stirred. After being left to equilibrate at (298, 323 or 353) K ± 1 K overnight, the two phases were carefully separated and weighed, allowing for the determination of the mentioned parameters. The pH of each phase was also determined. The fitting of the experimental binodal curves and calculation of the TLLs were accomplished according to the literature [44]. Studies on the ferulic acid partition were conducted using biphasic mixtures, and the experimental details can be found in the Supplementary Materials.

### 2.7. COSMO-RS

The purine-based IL σ-profile and hydrogen-bonding interaction energies (*E*_HB_) were estimated using COSMO-RS (conductor-like screening model for real solvents) thermodynamic model. Details about the employed methodology can be found elsewhere [45,46]. The quantum chemical COSMO calculations were performed with the TURBOMOLE 6.1 program package on the density functional theory (DFT) level, applying the BP functional B88-P86 with a triple-2 valence polarized basis set (TZVP) and the resolution of identity standard (RI) approximation [47]. The COSMOthermX program, using the parameter file BP_TZVP_C20_0111 (COSMOlogic GmbH & Co KG, Leverkusen, Germany), was used in all calculations [48].

## 3. Results and Discussion

Four novel purine-based salts, including three ILs, composed of tetrabutylammonium cation and the anions theobromine, theophylline, xanthine, and uric acid, were successfully synthesized in this work (Table 1). Aiming to develop fully bio-based compounds, the preparation of purine-based ILs with cholinium as a cation and theophylline as an anion was also tested, following the same synthesis protocol. It was not possible to obtain the aimed IL structure, but no protocol adaptations were carried out. Thus, with proper optimization, it could be possible to synthesize purine-based ILs with cholinium as a cation.

### 3.1. Synthesis and Characterization of Purine-Based ILs

The purine-based salts were obtained as solids at yields above 94% (see Table 1). The melting (T_m_) and degradation (T_d_) temperatures, as well as the melting enthalpy of [N_4444_][Theob], [N_4444_][Theop] [N_4444_][Xan], and [N_4444_][Ur], were determined by DSC and TGA (Table 2). The complexity of the [N_4444_][Xan] spectrum lead to the determination of its melting point using another melting point apparatus. Since [N_4444_][Ur] starts decomposing before melting, this compound is not categorized as an IL, according to the recent definition by Mariani et al. [3]. The melting temperature of [N_4444_][Theob] and [N_4444_][Theop] are very similar. The same does not occur for the purines with theobromine and theophylline, which have melting temperatures of 620 K and 544 K, respectively [49]. The high melting temperature of the purines is likely due to the strong intramolecular interactions between the N–H groups [50]. The introduction of the bulky [N_4444_]^+^ cation disrupts this efficient packing, lowering the lattice energy and melting point of the salts, in comparison to the purines. Nevertheless, the melting point of the purine-based ILs is still higher than the melting point of other conventional ILs, such as [N_4444_]Cl (344 K) [51]. As for the decomposition temperature of the synthesized salts, this property increased in the following order: [N_4444_][Theob] < [N_4444_][Theop] < [N_4444_][Xan] < [N_4444_][Ur] (Table 2). Theobromine and theophylline are known as methylxanthines and, in comparison to xanthine, they have two more methyl groups. Their lower T_d_ suggests that *N*-demethylation may be one of the pathways involved in these compounds degradation, causing [N_4444_][Theob] and [N_4444_][Theop] to degrade sooner than [N_4444_][Xan]. The degradation order of these ILs matches the order of the catabolic pathway of caffeine: caffeine → theobromine/theophylline → xanthine → uric acid [52]. This further supports the theory that *N*-demethylation is connected to the decomposition temperature values of the purine-based salts. This is not valid for uric acid, since the conversion of xanthine to uric acid occurs via oxidation. Still, [N_4444_][Ur] has the highest T_d_ value, while simultaneously being the last metabolite of the excerpt of this catabolic pathway.

**Figure 1:**
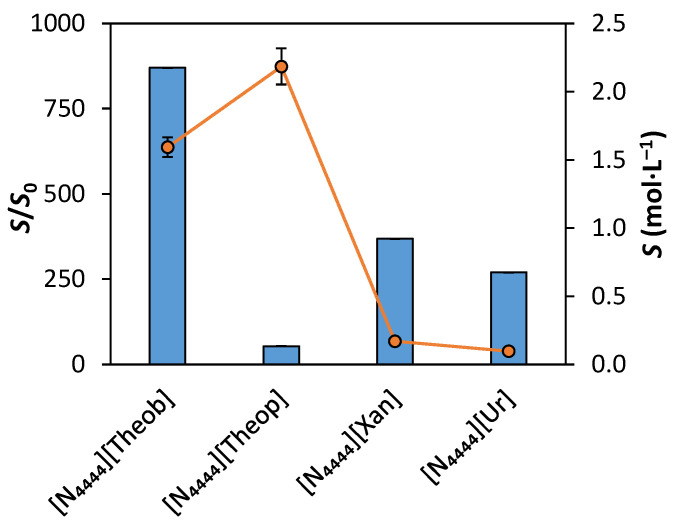
Aqueous solubility enhancement (*S*/*S*_0_) of each purine-based salt, relative to the respective free purine (blue bars) and molar solubility (*S*) of each salt in pure water (orange dots and line). *S*_0_ values were obtained from PubChem [53].

The aqueous solubility enhancement (*S*/*S*_0_), where *S* is the molar water-solubility of the purine-based salt and *S*_0_ is the molar water-solubility of its free corresponding purine, is depicted in Figure 1. Detailed data, including the pH of the saturated IL solutions, can be consulted in the Supplementary Materials, Table S1.

All the salts presented significant water-solubility improvements, when compared to the solubility of the purines, with the aqueous solubility enhancement ranging from 53- to 870-folds. The *S*/*S*_0_ of the purine-based salts increases as follows: [N_4444_][Theop] < [N_4444_][Ur] < [N_4444_][Xan] < [N_4444_][Theob]. Although [N_4444_][Theop] has the highest solubility value, its purine, theophylline, has a much higher solubility than the remaining evaluated purines (4.09 × 10^−2^ mol·L^−1^ vs. 3.57 × 10^−4^ mol·L^−1^ of uric acid as free purine), causing its solubility enhancement, *S/S*_0_, to be the lowest.

Regarding the solubility of each salt, this property increases in the following order: [N_4444_][Ur] < [N_4444_][Xan] < [N_4444_][Theob] < [N_4444_][Theop]. Although [N_4444_][Theob] and [N_4444_][Theop] are isomers, their solubility is significantly different. The same occurs in their free purines, with theobromine and theophylline having a water-solubility of 1.83 × 10^−3^ mol·L^−1^ and 4.09 × 10^−2^ mol·L^−1^, respectively [54]. Theobromine has relatively strong intermolecular hydrogen bonding between its carbonyl groups and strong π–π stacking interactions [18,55,56]. In theophylline, the hydrogen between the two carbonyl groups is replaced by a methyl group, causing the previously mentioned interactions to be weaker. Ultimately, this leads to theophylline being more soluble than theobromine [18,55,56], which is in agreement with the results obtained here. Still, the solubility difference between [N_4444_][Theop] and [N_4444_][Theob] is smaller than that recorded for their purines (see Table S1). In the ILs case, the anionic form of theobromine may be responsible for reducing the occurrence of π–π stacking, contributing to the higher solubility of the IL, when compared to its respective free purine. When comparing the structures of the four purine-based salt (Table 1) it is possible to conclude that both [N_4444_][Theob] and [N_4444_][Theop] have two more methyl groups than [N_4444_][Ur] and [N_4444_][Xan], as mentioned before. Adding alkyl groups to ILs usually lowers their solubility [57]. However, this was not observed in the present work. The overall poor solubility of purines is connected to their inter-base hydrogen bonding [56]. The insertion of the methyl groups replaces protons that would otherwise be available to form inter-base hydrogen bonds [56]. Consequently, the insertion of the two methyl groups increases the solubility of the methylated purines, in comparison to the non-methylated ones. This is in agreement with our results, with [N_4444_][Theob] and [N_4444_][Theop] being more soluble than [N_4444_][Xan] and [N_4444_][Ur]. Regarding [N_4444_][Ur], this salt also afforded better water-solubility (0.096 ± 0.007 mol·L^−1^) than other urate salts, such as sodium urate (0.00676 mol·L^−1^) and potassium urate (0.01206 mol·L^−1^) [58]. To better understand the solubility improvements of the ILs and urate-based salt, a simple test was performed by saturating an aqueous solution of [N_4444_]Cl with sodium theophyllinate and evaluating the latter’s water-solubility (see the Supplementary Materials, Table S2). Sodium theophyllinate has better water-solubility than theophylline, but its solubility is not improved in aqueous solutions of [N_4444_]Cl. This shows that the solubility improvements seen here are due to the formation of the salt, rather than the hydrotropic effect of the cation.

**Figure 2:**
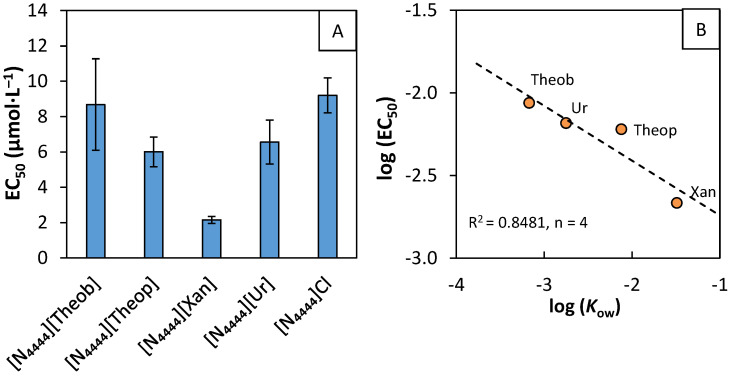
(**A**) EC_50_ values (μmol∙L^−1^), determined after 96 h of exposure time of *Raphidocelis subcapitata* to the purine-based salts and [N_4444_]Cl. The error bars correspond to the 95% confidence interval. (**B**) Correlation between the logarithm of EC_50_ and the logarithm of the octanol–water partition coefficient (*K*_ow_) of the different salt anions [59]. The coefficient of determination (*R*^2^) and the number of experimental points used for the linear regression (*n*) are also presented.

The ecotoxicity of the synthesized purine-based salts and [N_4444_]Cl was determined using the microalgae *Raphidocelis subcapitata*. The obtained EC_50_ values (μmol∙L^−1^), after 96 h of incubation, and their correlation with the octanol–water partition coefficient of the salt anions is shown in Figure 2. The EC_50_ values in mg∙L^−1^ are available for consultation in Supplementary Materials, Table S3.

According to the classifications attributed by the European legislation [60], all the evaluated salts fit in the acute 2 category (1 < EC_50_ < 10 mg∙L^−1^), except for [N_4444_][Xan], which belongs in the acute 1 category (EC_50_ < 1 mg∙L^−1^)–consult See Supplementary Materials for more details on salts EC_50_ values in mg∙L^−1^. The salts ecotoxicity increases in the following order: [N_4444_]Cl < [N_4444_][Theob] < [N_4444_][Ur] < [N_4444_][Theop] < [N_4444_][Xan]. According to Egorova et al. [61], the ecotoxicity of ILs is mostly influenced by the alkyl chain length and side-chain functionalization of the cation, both the cation and anion nature, and the mutual influence of the anion–cation combination. In our work, the anion has little impact on the ecotoxicity of the ILs, suggesting that the ecotoxicity is dominated by the cation, which is in close agreement with other reports [62,63]. The tetrabutylammonium cation has four butyl side-chains, linked to a central heteroatom, causing it to have a higher hydrophobic profile. As a consequence, this cation is more prone to interact with the hydrophobic domains present in the microalgae’s cell wall and potentially cause it to disrupt [63]. Although the anion has a small effect on the salts ecotoxicity, it is not insignificant. It was found that purine-based salts ecotoxicity is correlated with the logarithm of their anion octanol–water partition coefficient, log (*K*_ow_) (Figure 2B). The obtained results suggest that salts, composed of anions with lower log (*K*_ow_) values, and, thus, a higher affinity for water, have lower ecotoxicity values, which is in agreement with the literature [64]. However, [N_4444_]Cl ecotoxicity is out of this trend.

The incorporation of naturally occurring purines as anions was not sufficient to balance the ILs and salt ecotoxicity. As also shown by other reports, employing biocompatible feedstock for the design of ILs does not always translate into eco-friendly ILs [65,66]. There is no simple answer for the synthesis of biocompatible ILs, which reinforces the need to do (eco)toxicity screening evaluations and consequent structure optimizations, aiming to achieve more benign ILs.

### 3.2. Aqueous Biphasic Systems Formation

**Figure 3:**
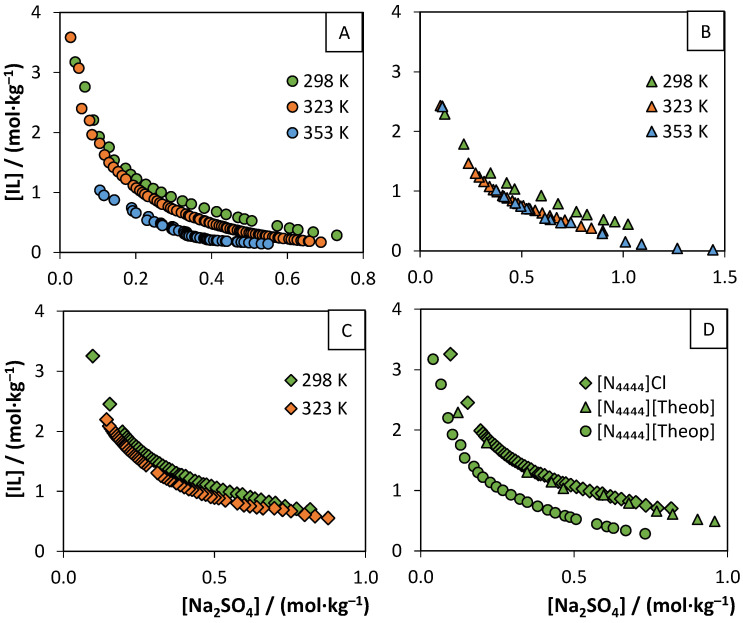
Binodal curves of the ternary systems, composed of (**A**) [N_4444_][Theop], (**B**) [N_4444_][Theob], or (**C**) [N_4444_]Cl, water, and Na_2_SO_4_ at 298 K (green), 323 K (orange), or 353 K (blue) (±1 K) and atmospheric pressure (0.1 MPa). [N_4444_]Cl was not tested at 353 K. (**D**) Comparison of the binodal curves of [N_4444_][Theop] (o), [N_4444_][Theob] (Δ), or [N_4444_]Cl (◊) at 298 K, using Na_2_SO_4_ as a salting-out agent.

**Figure 4:**
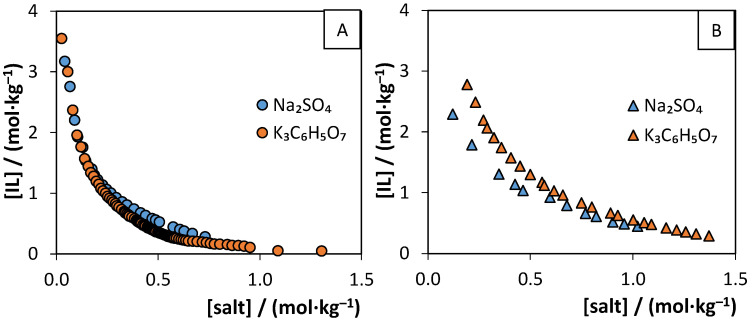
Binodal curves of the ternary systems, composed of (**A**) [N_4444_][Theop] or (**B**) [N_4444_][Theob], water, and Na_2_SO_4_ (blue) or K_3_C_6_H_5_O_7_ (orange) at (298 ± 1) K and atmospheric pressure (0.1 MPa).

To unlock new applications for the water-soluble purine-based salts synthesized in this work, their ability to form ABS was evaluated. The lower solubility of [N_4444_][Ur] (0.096 ± 0.007 mol∙L^−1^) and [N_4444_][Xan] (0.17 ± 0.01 mol∙L^−1^) prevented their application in the formation of ABS. Still, the ternary phase diagrams of [N_4444_][Theob], [N_4444_][Theop], and [N_4444_]Cl ILs with Na_2_SO_4_ and K_3_C_6_H_5_O_7_ as salting-out agents were determined. To evaluate the potential ability to form thermoresponsive ABS, three different temperatures were studied in the ILs-Na_2_SO_4_ systems: 298, 323, and 353 (±1) K. The determined binodal curves, using Na_2_SO_4_ as salting-out agents, are shown in Figure 3 and Supplementary Materials, Figure S2. The comparison of binodal curves using different salts (Na_2_SO_4_ and K_3_C_6_H_5_O_7_) at 298 K is depicted in Figure 4. The experimental weight fraction data, Merchuk equation parameters, and TL data are reported in the Supplementary Materials, Tables S4–S11. The biphasic region is located above the binodal curve.

**Figure 5:**
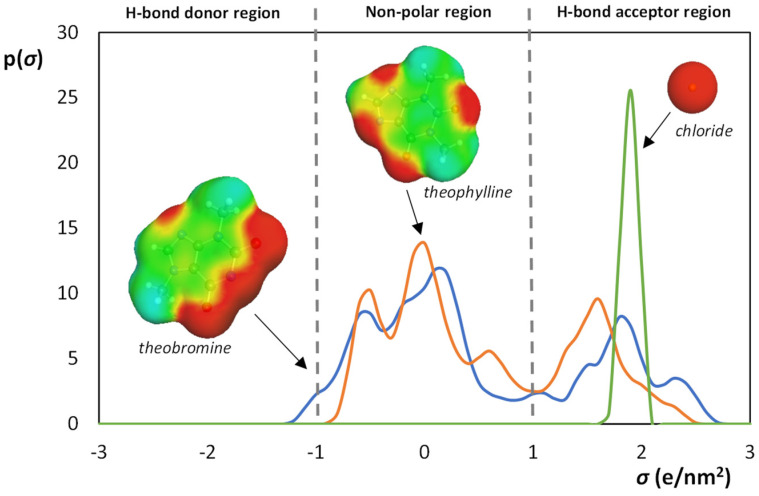
σ-Profiles and surfaces of ILs anions. Different ILs are represented in different colors: [N_4444_][Theop] (orange), [N_4444_][Theob] (blue), [N_4444_]Cl (green). The molecular surface charge distribution is represented in red (polar segments), blue (apolar segments), and green (neutral segments).

The ILs ability to form ABS increases as follows: [N_4444_]Cl < [N_4444_][Theob] < [N_4444_][Theop]. Among the evaluated ILs, [N_4444_][Theop] showed the best ability to form ABS and the most significant thermoresponsive behavior. Although [N_4444_][Theob] and [N_4444_][Theop] have a strong structural similarity, their ability to induce demixing is different. The demixing capacity is ruled by the competition of all ions for the formation of hydration complexes [67]. This correlates with both the hydrogen-bond basicity of the IL and the molar entropy of the salt. High charge density salts are more prone to establish water hydration complexes, inducing the formation of the ABS, by salting-out the IL from the aqueous media. ILs with higher affinity for water and, therefore, higher hydrogen-bond basicity, difficult their salting-out and, consequently, ABS formation. To better understand the obtained experimental results, the σ-profiles and hydrogen-bonding interaction energies (*E*_HB_) of each IL were determined using COSMO-RS. The σ-profiles and surfaces of the IL anions are presented in Figure 5. As expected, the σ-profiles of the theophylline and theobromine anions are similar, presenting the main peaks at the non-polar and H-bond acceptor regions, while chloride anion has a single peak at the H-bond acceptor region. In what concerns the hydrogen-bonding interaction energies of the studied ILs, the *E*_HB_ increased as follows: −6.45 kJ [N_4444_][Theob] < −4.51 kJ [N_4444_]Cl < −3.55 kJ [N_4444_][Theop]. ILs with more negative *E*_HB_ values are more prone to accept protons and, thus, to interact with water [67]. As seen by the presented values, [N_4444_][Theob] has the most negative *E*_HB_ value, meaning that the liquid–liquid demixing occurs at higher IL concentrations than for [N_4444_][Theop]. This is not so straightforward for [N_4444_]Cl. Although the binodal curves of [N_4444_]Cl and [N_4444_][Theob] are similar (Figure S2, Supplementary Materials), their *E*_HB_ values are considerably different.

Experimental results, presented in Figure 4, show that the salt influence on the formation of the ABS depends on the IL. In systems using [N_4444_][Theop], the binodal curves are similar, with K_3_C_6_H_5_O_7_ being a slightly better ABS inducer than Na_2_SO_4_. The same does not apply for [N_4444_][Theob] binodal curves, with Na_2_SO_4_ being a better ABS promoter than K_3_C_6_H_5_O_7_. This change in tendency was also observed with other ILs [67]. Take the formation of ABS with [N_4444_]Cl/[N_4444_]Br and Na_2_SO_4_/K_3_C_6_H_5_O_7_ as an example, for which the K_3_C_6_H_5_O_7_ salt ability to form ABS with [N_4444_]Br is slightly better than the one reported for Na_2_SO_4_ [68]. However, the anion change from bromide to chloride leads to the modification of this tendency, with K_3_C_6_H_5_O_7_ being a stronger salting-out agent than Na_2_SO_4_ [67,68]. Overall, the salt seems to have a small impact on the binodal curves, under the tested conditions.

As for the thermoresponsive feature of the systems, all ILs show different behaviors: [N_4444_][Theop] is sensitive to all temperature variations, forming three distinctive systems; [N_4444_][Theob] responds to the temperature shift from 298 K to 323 K, but the binodal curves at 323 K and 353 K are overlapping. [N_4444_]Cl has a smaller response to the tested temperature stimulus. Altogether, the purine-based ILs seem to have better thermoresponsive behavior than [N_4444_]Cl. All the systems present a lower critical solution temperature (LCST) phase transition type, meaning that the formation of the ABS is favored by the increase of temperature. In addition, the thermoresponsive behavior of the ABS was found to be reversible. If a monophasic ternary mixture at 298 K is heated up to 323 or 353 K, a biphasic ternary mixture is formed. However, when that mixture is cooled down to 298 K, the system turns monophasic again. The thermoresponsive character of these systems unlocks more potential applications of the purine-based ILs, enabling not only the extraction and separation of molecules but also their recovery and reuse [69]. The [N_4444_][Theob]-Na_2_SO_4_-water system is particularly promising, since smaller temperature increases (from 298 to 323 K) lead to larger differences in the binodal curves. Although the thermoresponsive behavior of these ILs is less pronounced than in other reported ILs [70], the LCST behavior is usually found in IL–polymer–water systems and not in IL–salt–water systems [69,70]. Herein, the ability to form thermoresponsive systems using Na_2_SO_4_ as a salting-out agent may be related to the self-aggregation of the tetrabutylammonium cation [71]. Tie-lines (TL) were determined, as detailed in the Supplementary Materials (in Tables S10 and S11).

To further reinforce the relevance of purine-based ILs application in the formation of ABS, a brief study on the ability to partition a biomolecule (ferulic acid), using mixture points from the determined systems, was conducted at 298 K (data detailed in the Supplementary Materials, Figure S3). The purine-based ILs and [N_4444_]Cl had a similar extraction performance, with 96 to 100% of ferulic acid being extracted to the top phase, regardless of the used salting-out agent (K_3_C_5_H_6_O_7_ or Na_2_SO_4_).

### 3.3. Solubility Enhancement in Aqueous Solutions

**Figure 6:**
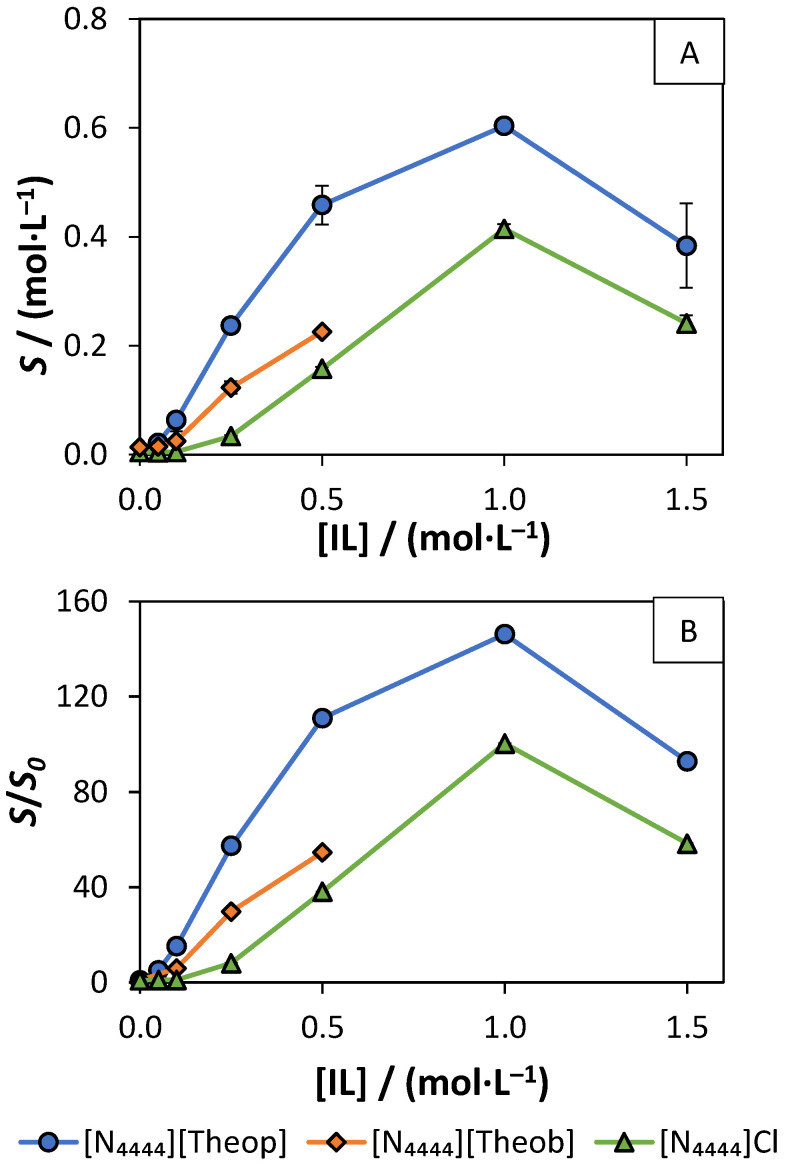
(**A**) Molar solubility of ferulic acid in IL solutions with different concentrations. (**B**) Molar aqueous solubility enhancement (*S*/*S*_0_) of ferulic acid in IL solutions with different concentrations. In both (**A**) and (**B**), different ILs are represented in different colors: [N_4444_][Theop] (blue o), [N_4444_][Theob] (orange ◊), and [N_4444_]Cl (green Δ).

To study the solubility enhancement capacity of the purine-based ILs, aqueous solutions of [N_4444_][Theop] and [N_4444_][Theob] were prepared at different molar concentrations, and ferulic acid was used as a poorly water-soluble solute. The lower solubility of [N_4444_][Ur] and [N_4444_][Xan] prevented the study of their solubility enhancement capacity. [N_4444_]Cl solubility enhancement capacity was also studied, in order to compare its efficiency with the purine-based ILs. The molar solubility and the molar aqueous solubility enhancement (*S*/*S*_0_) of ferulic acid at different concentrations of IL are depicted in Figure 6, and detailed data can be consulted in the Supplementary Materials (Table S12). The *S*_0_ value (0.0041 ± 0.0002 mol∙L^−1^) was obtained by saturating ferulic acid in pure water, which is in agreement with the reported ferulic acid water-solubility of 0.00470 ± 0.00001 mol∙L^−1^ [72]. The IL integrity was confirmed by ^1^H and ^13^C NMR. No data is presented for [N_4444_][Theob] at 1.0 and 1.5 mol∙L^−1^ since ionic exchange was verified at these IL concentrations.

**Figure 7:**
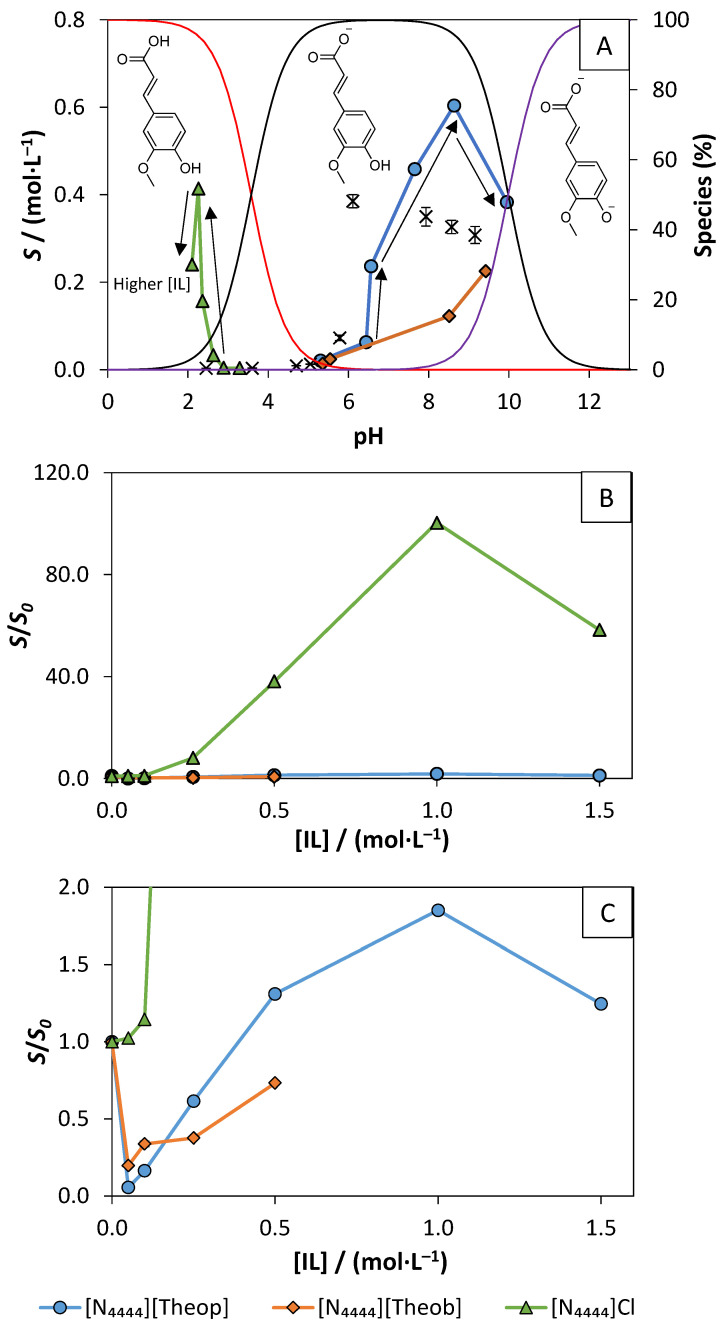
(**A**) Ferulic acid molar solubility, left axis, throughout the pH of the saturated samples with IL concentrations ranging from 0.05 to 1.5 mol∙L^−1^: water-solubility of ferulic acid at that pH (×); [N_4444_][Theop] (blue o), [N_4444_][Theob] (orange ◊), and [N_4444_]Cl (green Δ). The red, black, and purple curves represent the deprotonation percentage of ferulic acid, right axis (data from Marvin 21.14 [59]). The arrows point to IL concentration increase. (**B**) Molar aqueous solubility enhancement (*S*/*S*_0_) of ferulic acid in [N_4444_][Theop] (blue ○), [N_4444_][Theob] (orange ◊), and [N_4444_]Cl (green Δ) solutions at different molar concentrations, with S_0_ being the water-solubility of ferulic acid at a similar pH to that of the saturated sample; (**C**) *y*-axis zoom of (**B**).

The molar solubility of ferulic acid seems to improve in the following order: [N_4444_]Cl < [N_4444_][Theob] < [N_4444_][Theop]. At lower IL concentrations (the diluted region), it is possible to calculate the Setschenow constant, which correlates the ratio between the solubility enhancement and solubility enhancer concentration in the diluted region. The solubility data obtained at the diluted region was used to calculate the Setschenow constant, according to the following equation [73]:

(1) $$\ln\left(S\right)=K_{HYD}\times C_{IL}+\ln\left(S_{0}\right)$$

where *S* is the molar solubility of ferulic acid, *K_HYD_* is the empirical Setschenow constant, *C_IL_* is the molar concentration of the IL, and *S*_0_ is the water-solubility of ferulic acid. At lower IL concentrations, the solubility enhancement of ferulic acid, imposed by the different ILs presence, increases in the following sequence: [N_4444_]Cl < [N_4444_][Theob] < [N_4444_][Theop], which is further corroborated by the values of their Setschenow constants of 2.66 ([IL] ≤ 0.10 mol∙L^−1^), 9.44 ([IL] ≤ 0.25 mol∙L^−1^), and 11.71 L∙mol^−1^ ([IL] ≤ 0.25 mol∙L^−1^), respectively (see Figure S4 and Table S13). Regarding the *S*/*S*_0_ of ferulic acid in IL solutions, all the ILs seem to enhance the solubility of ferulic acid (up to 146-folds). Yet, the pH-dependency of the solubility of ferulic acid was not taken into consideration in the previously presented data. To understand how pH influences the obtained solubility results, the water-solubility of ferulic acid at different pH values and pH of the saturated samples were studied. The pH of the samples was greatly dependent on the IL nature and its concentration (see Figure 7A and Table S14 in the Supplementary Materials). Since the solubility of ferulic acid depends on pH, different *S*_0_ values were used to determine the *S*/*S*_0_, according to the pH value of the samples (Figure 7B and Table S15 in the Supplementary Materials).

The IL solutions saturated with ferulic acid have different pH values over the tested IL concentrations range (Supplementary Materials-Table S14). The addition of ferulic acid to the IL solutions lowered their pH, with this change being more evident in solutions with lower IL concentrations. Controlling the pH of the samples would require the addition of buffers, which would interfere with the solubility enhancement assays. For this reason, no attempts were made to adjust the pH values of the samples, and, for each sample, the initial and final pH values were registered. In the [N_4444_]Cl solutions, the pH of the samples was similar to the different IL concentrations, ranging from 2.10 to 3.29. In this case, ferulic acid was in its neutral form and [N_4444_]Cl improved its solubility (up to 117-folds) through hydrotropy in a pH-independent way (Figure 7A). For [N_4444_][Theob] and [N_4444_][Theop], a wide range of pH values were obtained, depending on the IL concentration. For [N_4444_][Theob] and [N_4444_][Theop], the pH of the samples increases as the IL concentration increases (5.36 to 9.62 and 5.30 to 9.95, respectively), leading to the deprotonation of ferulic acid. When ferulic acid is deprotonated, its solubility is mostly ruled by pH, rather than hydrotropy. Due to the impact of pH in the solubility of ferulic acid, the *S*/*S*_0_ was recalculated using the ferulic acid water-solubility at the most suitable pH, i.e., using a *S*_0_ value determined to be at a similar pH value to that of the sample. To do so, the water-solubility (*S*_0_) of ferulic acid was determined at different pH values (consult Table S15 in the Supplementary Materials). Since the addition of ferulic acid significantly lowers the pH of the aqueous solutions, it was necessary to add NaOH to the aqueous solutions, in order to measure its water-solubility at high pH values. The *S*/*S*_0_ recalculation (Figure 7C) showed that the solubility enhancement of ferulic acid, by [N_4444_][Theob], is mainly ruled by pH, being its solubility improvement lower than the one provided by pH alone (*S*/*S*_0_ < 1). Regarding [N_4444_][Theop], at IL concentrations ranging from 0.5 to 1.5 mol∙L^−1^, the ferulic acid solubility enhancement is higher than the one caused by pH (*S*/*S*_0_ > 1). This means that [N_4444_][Theop] has a hydrotropic effect besides the pH-dependent solubility enhancement. The ferulic acid solubility seems to achieve its maximum peak at IL concentrations of 1.0 mol∙L^−1^.

Charged molecules are more soluble than neutral ones. Thus, ferulic acid speciation changes from 0 to −1 or −2 global net charge, results in higher *S*_0_ values and a lower hydrotropic effect, causing pH to have a great influence on the solubility of charged molecules. However, this does not justify why the hydrotropic effect of the tetrabutylammonium cation significantly dropped in the samples containing higher pH values. The hydrotropic behavior of the tetrabutylammonium cation has been reported towards other molecules [4,32], suggesting that other effects may be influencing the solubility of ferulic acid at higher pH values. Briefly, despite pH having a great impact on the solubility of ferulic acid, [N_4444_][Theop] was still able to demonstrate a hydrotropic effect at IL concentrations ranging from 0.5 to 1.5 mol∙L^−1^, increasing the solubility of ferulic acid up to two-folds. As a side note, it should be highlighted that the purine-based ILs enable an effortless solubilization of ferulic acid at basic pH values, which could be advantageous for potential applications requiring ferulic acid to be at alkaline pH values.

## 4. Conclusions

Three purine-based ILs and one salt having tetrabutylammonium as a cation, were synthesized and characterized, regarding their melting and degradation temperatures, water-solubility, and ecotoxicity against the green microalgal *Raphidocelis subcapitata*. The synthesized salts displayed good thermal stability, decomposing from 457 to 505 K. *N*-demethylation seems to play a role in the thermal decomposition of the purine-based ILs. In comparison to their purine precursors, the purine-based ILs afforded much higher aqueous solubilities, ranging from 53- for [N_4444_][Theop] to 870-fold for [N_4444_][Theob]. The [N_4444_][Theop] had the higher solubility, with a value of 2.2 ± 0.1 mol∙L^−1^ vs. the 0.096 ± 0.007 mol∙L^−1^ of the least soluble compound, [N_4444_][Ur]. Although all the synthesized salts have bio-based anions, their ecotoxicity performance is not very good. Ecotoxicity was dominated by the cation which impact is greater than that of the anion, causing none of the salts to be deemed as non-hazardous. All the synthesized compounds and [N_4444_]Cl belong in the acute 2 category (1 < EC_50_ < 10 mg∙L^−1^), apart from [N_4444_][Xan], which belongs in the acute 1 category (EC_50_ < 1 mg∙L^−1^). The solubility of [N_4444_][Xan] and [N_4444_][Ur] was not sufficient to enable the formation of ABS. However, the remaining purine-based ILs were able to form ABS with both sodium sulfate and tripotassium citrate. The thermoresponsive character of the ABS was evaluated using the systems salted-out with sodium sulfate at 298, 323, and 353 (± 1) K and atmospheric pressure. [N_4444_][Theop] showed the best response to the thermal stimulus, while [N_4444_][Theob] showed a thermoresponse from temperature shifts of 298 to 323 or 353 (± 1) K but not from the temperature shift of 323 to 353 (± 1) K. The [N_4444_]Cl showed a less accentuated thermal response at the tested temperatures. Finally, the hydrotropic effect of the purine-based ILs and [N_4444_]Cl was evaluated. While [N_4444_]Cl acted as an hydrotrope in a pH-independent way, [N_4444_][Theob] and [N_4444_][Theop] enhanced the solubility of ferulic acid in a highly pH-dependent way.

## Figures and Tables

**Table 1:** Synthesized salts and respective names, abbreviations, chemical structures, and synthesis yields (%).

Name/Abbreviation	Structure	Yield (%)
Tetrabutylammonium theobrominate [N_4444_][Theob]	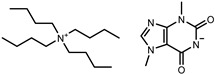	100
Tetrabutylammonium theophyllinate [N_4444_][Theop]	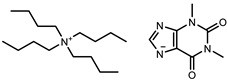	97
Tetrabutylammonium xanthinate [N_4444_][Xan]	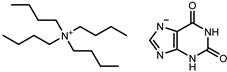	94
Tetrabutylammonium urate [N_4444_][Ur]	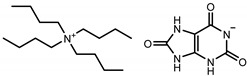	97

**Table 2:** Melting temperature (T_m_), melting enthalphy ΔH_m_, and decomposition temperature (T_d_) of the purine-based salts.

Salt	T_m_/(K)	ΔH_m_/(J·mol^−1^)	T_d_/(K)
[N_4444_][Theob]	377.8 ± 0.2	50920 ± 51	457 ± 1
[N_4444_][Theop]	370.6 ± 0.1	36853 ± 1811	486 ± 1
[N_4444_][Xan]	487 ± 1	- ^a^	495 ± 1
[N_4444_][Ur]	- ^a^	- ^a^	505 ± 1

^a^ Not possible to determine.

## Data Availability

Not applicable.

## Supplementary Materials

The following are available online. Spectrum S1, ^1^H NMR spectra of [N_4444_][Theob] (D_2_O, 300 MHz); spectrum S2, ^13^C NMR spectra of [N_4444_][Theob] (D_2_O, 75.47 MHz); spectrum S3, ^1^H NMR spectra of [N_4444_][Theop] (D_2_O, 300 MHz); spectrum S4, ^13^C NMR spectra of [N_4444_][Theop] (D_2_O, 75.47 MHz); spectrum S5, ^1^H NMR spectra of [N_4444_][Xan] (D_2_O, 300 MHz); spectrum S6, ^13^C NMR spectra of [N_4444_][Xan] (D_2_O, 75.47 MHz); spectrum S7, ^1^H NMR spectra of [N_4444_][Ur] (D_2_O, 300 MHz); spectrum S8, ^13^C NMR spectra of [N_4444_][Ur] (D_2_O, 75.47 MHz); Figure S1, ferulic acid absorbance spectrum in an aqueous solution of [N_4444_][Theob] at 0.5 mol∙L^−1^; Table S1, molar water-solubility (*S*) of the purine-based ILs, pH of the saturated IL solution at (298 ± 1) K, and molar water-solubility of their respective purines (*S*_0_); Table S2, molar solubility (*S*) of sodium theophyllinate in an aqueous solution of [N_4444_]Cl (1.0 mol∙L^−1^), in pure water (*S*_0_), and its molar aqueous solubility enhancement (*S*/*S*_0_). The pH of the initial aqueous solution of [N_4444_]Cl and final saturated solution are also shown; Table S3, EC_50_ values (mg∙L^−1^) of the purine-based ILs and [N_4444_]Cl, after 96 h of incubation with the microalgae Raphidocelis subcapitata and the logarithm function of their anion octanol–water partition coefficient, log (*K*_ow_). The respective 95% confidence limits are presented in the parenthesis; Figure S2, binodal curves of the ternary systems, composed of [N_4444_][Theop] (blue), [N_4444_][Theob] (orange), or [N_4444_]Cl (green), water and Na_2_SO_4_ at 298 K (Δ), 323 K (○), or 353 K (◇) (±1 K), as well as atmospheric pressure (0.1 MPa). [N_4444_]Cl was not tested at (353 ± 1) K; Table S4, experimental weight fraction of the ternary systems composed of IL, water, and Na_2_SO_4_ at (298 ± 1) K and atmospheric pressure (0.1 MPa); Table S5, experimental weight fraction of the ternary systems, composed of IL, water, and Na_2_SO_4_ at (323 ± 1) K, as well as atmospheric pressure (0.1 MPa); Table S6, experimental weight fraction of the ternary systems composed of IL, water, and Na_2_SO_4_ at (353 ± 1) K; Table S7, experimental weight fraction of the ternary systems, composed of IL, water, and K_3_C_6_H_5_O_7_ at (298 ± 1) K; Table S8, Merchuk equation parameters (A, B, and C) and the respective standard deviations (σ) for the ternary systems composed of IL, water and Na_2_SO_4_ at 298 K, 323 K and 353 K (±1 K); Table S9. Merchuk equation parameters (A, B and C) and the respective standard deviations (σ) for the ternary systems composed of IL, water, and K_3_C_5_H_6_O_7_ at (298 ± 1) K; Table S10, experimental TLs and TLLs of the ternary systems composed of ILs + Na_2_SO_4_ at 298 K or 323 K (±1 K) and atmospheric pressure (0.1 MPa). No TLs were determined at (353 ± 1) K; Table S11, experimental TLs and TLLs of the ternary systems composed of ILs + K_3_C_5_H_6_O_7_ at (298 ± 1) K and atmospheric pressure (0.1 MPa); Figure S3, extraction efficiency (*EE*%) of each ternary mixture at (289 ± 1) K and atmospheric pressure (0.1 MPa). Blue bars represent data from systems containing the salting-out agent K_3_C_5_H_6_O_7_, and orange bars represent ternary mixtures with Na_2_SO_4_ as the salting-out agent; Table S12, molar solubility (*S*) of ferulic acid in IL solutions with different concentrations; Figure S4, fitted regression to the diluted region solubility data for the determination of the Setschenow constant. Different ILs are represented in different colors: [N_4444_][Theop] (blue), [N_4444_][Theob] (orange), and [N_4444_]Cl (green); Table S13, Setchenow constant for each IL, given by the slope of the fitted regression to the diluted region solubility data and the respective coefficient of determination (*R*^2^); Table S14, measured pH of the IL solutions, before the addition of ferulic acid and after obtaining a saturated solution; Table S15, ferulic acid water-solubility at different pH values.

## Sample Availability

Samples of the compounds are not available from the authors.
