# Supplementary material for: Synthesis of Purine-Based Ionic Liquids and Their Applications
Source: Molecules. 2021 Nov 18;26(22):6958. doi: 10.3390/molecules26226958 (PMC8620494; doi:10.3390/molecules26226958)
Supplement: Supplementary file 1 [file molecules-26-06958-s001.zip › molecules-1438583-supplementary.pdf]

# Synthesis of purine-based ionic liquids and their applications

Ana R. F. Carreira,[a] Telma Veloso,[a,b] Nicolas Schaeffer,[a] Joana L. Pereira,[b] Sónia P. M. Ventura,[a] Cécile Rizzi,[c] Juliette Sirieix Plénet,[c] Helena Passos,[a*] João A. P. Coutinho[a]

[a]CICECO - Aveiro Institute of Materials, Department of Chemistry, University of Aveiro, 3810-193 Aveiro, Portugal

[b]Department of Biology & CESAM, University of Aveiro, Aveiro, Portugal

[c]Sorbonne Université, Laboratoire Physico-Chimie des Électrolytes et Nano-Systèmes Interfaciaux, PHENIX,-UMR 8234, F-75252, Paris Cedex 05, France

*Corresponding author. Email: hpassos@ua.pt

**Nuclear magnetic resonance (NMR) characterization**

**Tetrabutylammonium theobrominate, [$N_{4444}$][Theob]:**

$^1$H NMR ($D_2O$, 300 MHz, [ppm]): δ 0.78 (t, $J_{HH}$ = 7.3 Hz, 12H, $N^+CH_2CH_2CH_2\boldsymbol{CH}_3$), 1.19 (sext, $J_{HH}$ = 7.3 Hz, 8H, $N^+CH_2CH_2\boldsymbol{CH}_2CH_3$), 1.37 – 1.57 (m, 8H, $N^+CH_2\boldsymbol{CH}_2CH_2CH_3$), 2.89 – 3.12 (m, 8H, $N^+\boldsymbol{CH}_2CH_2CH_2CH_3$), 3.24 (s, 3H, -$CONC\boldsymbol{H}_3$-), 3.71 (s, 3H, -$NCHC\boldsymbol{H}_3$-), 7.49 (s, 1H, -$NC\boldsymbol{H}N$-). $^{13}$C NMR ($D_2O$, 75.47 MHz, [ppm]): δ 12.8, 19.1, 23.0, 29.3, 33.2, 58.0, 109.6, 141.2, 149.9, 159.4, 165.1.

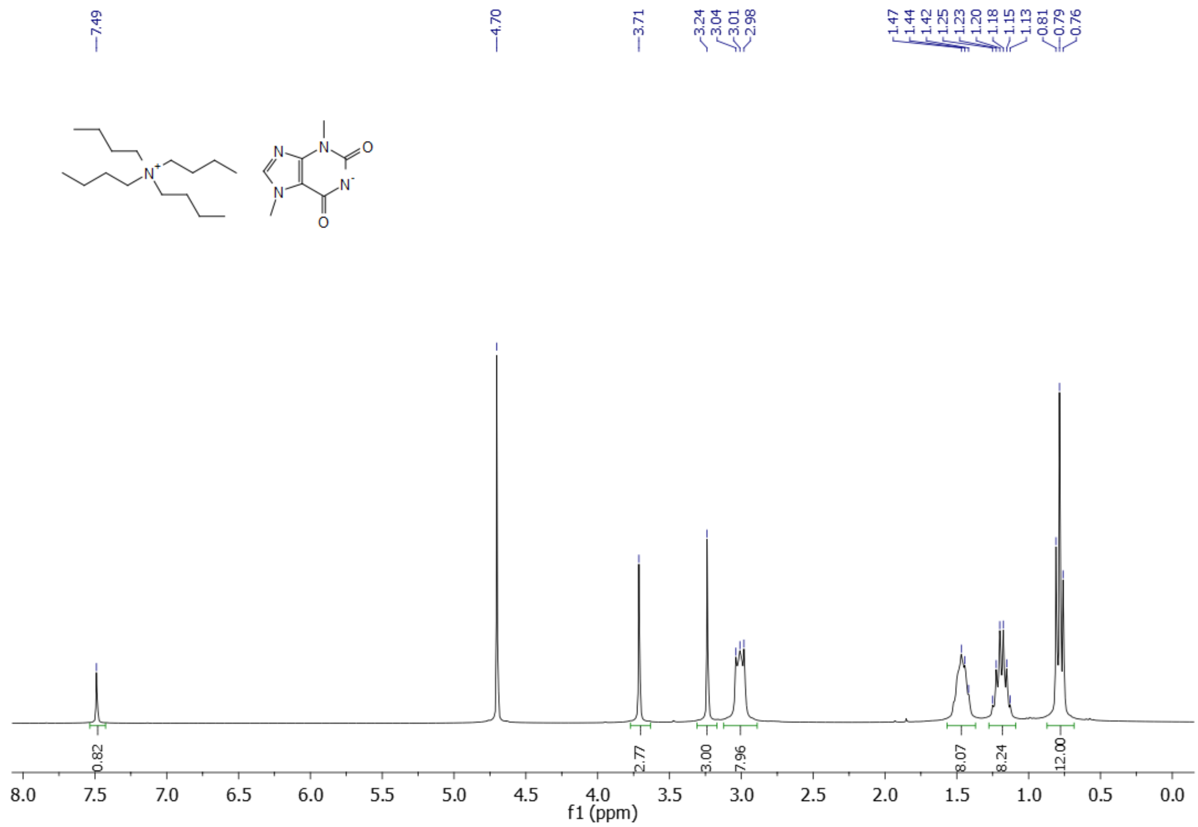


**Spectrum S1.** $^1$H NMR spectra of [$N_{4444}$][Theob] ($D_2O$, 300 MHz).

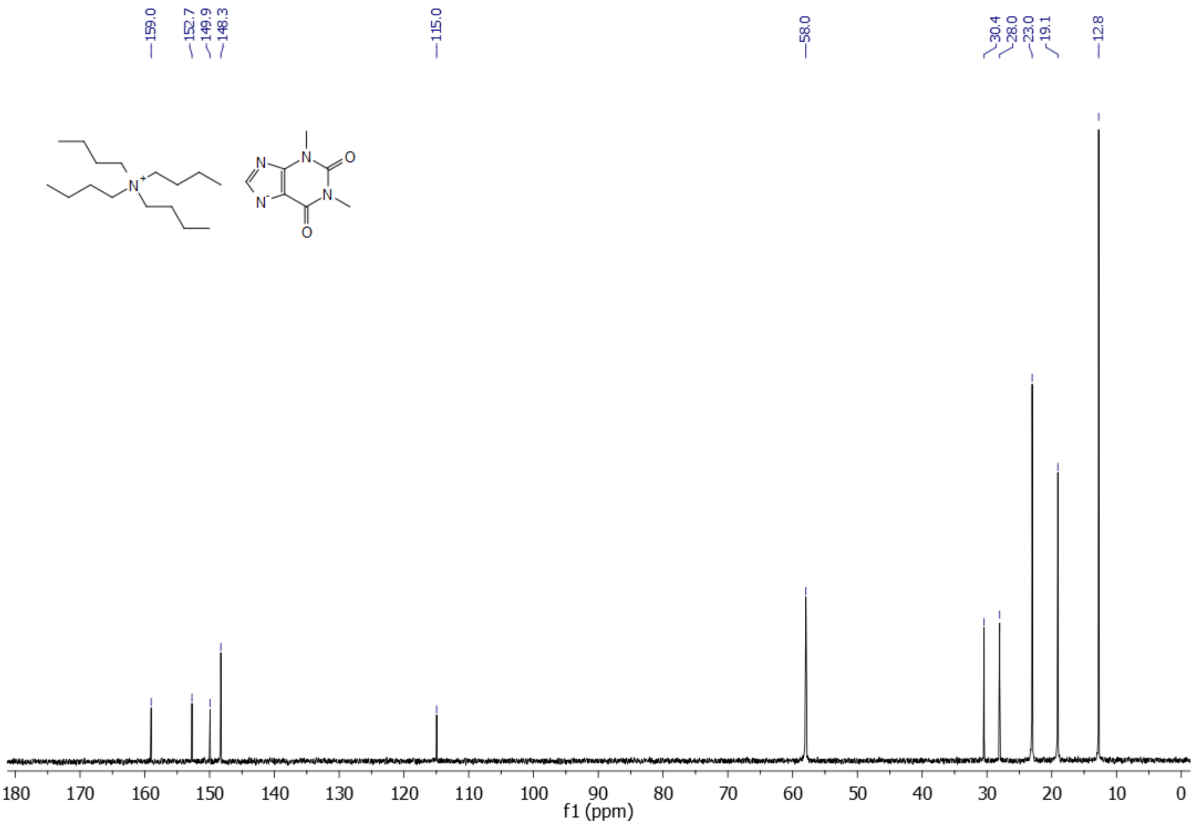


**Spectrum S2.** $^{13}$C NMR spectra of [$N_{4444}$][Theob] ($D_2O$, 75.47 MHz).

**Tetrabutylammonium theophillinate, [$N_{4444}$][Theop]:**

$^{1}$H NMR ($D_2O$, 300 MHz, [ppm]): δ 0.80 (t, $J_{HH}$ = 7.3 Hz, 12H, $N^+CH_2CH_2CH_2\boldsymbol{CH_3}$), 1.20 (sext, $J_{HH}$ = 7.3 Hz, 8H, $N^+CH_2CH_2\boldsymbol{CH_2}CH_3$), 1.23 – 1.51 (m, 8H, $N^+CH_2\boldsymbol{CH_2}CH_2CH_3$), 2.95 – 3.01 (m, 8H, $N^+\boldsymbol{CH_2}CH_2CH_2CH_3$), 3.20 (s, 3H, -CON(C$\boldsymbol{H_3}$)CO-), 3.36 (s, 3H, -CON(C$\boldsymbol{H_3}$)C-), 7.39 (s, 1H, -NC***H***N-). $^{13}$C NMR ($D_2O$, 75.47 MHz, [ppm]): δ 12.8, 19.1, 23.0, 28.0, 30.4, 58.0, 115.0, 148.3, 149.9, 152.7, 159.0.

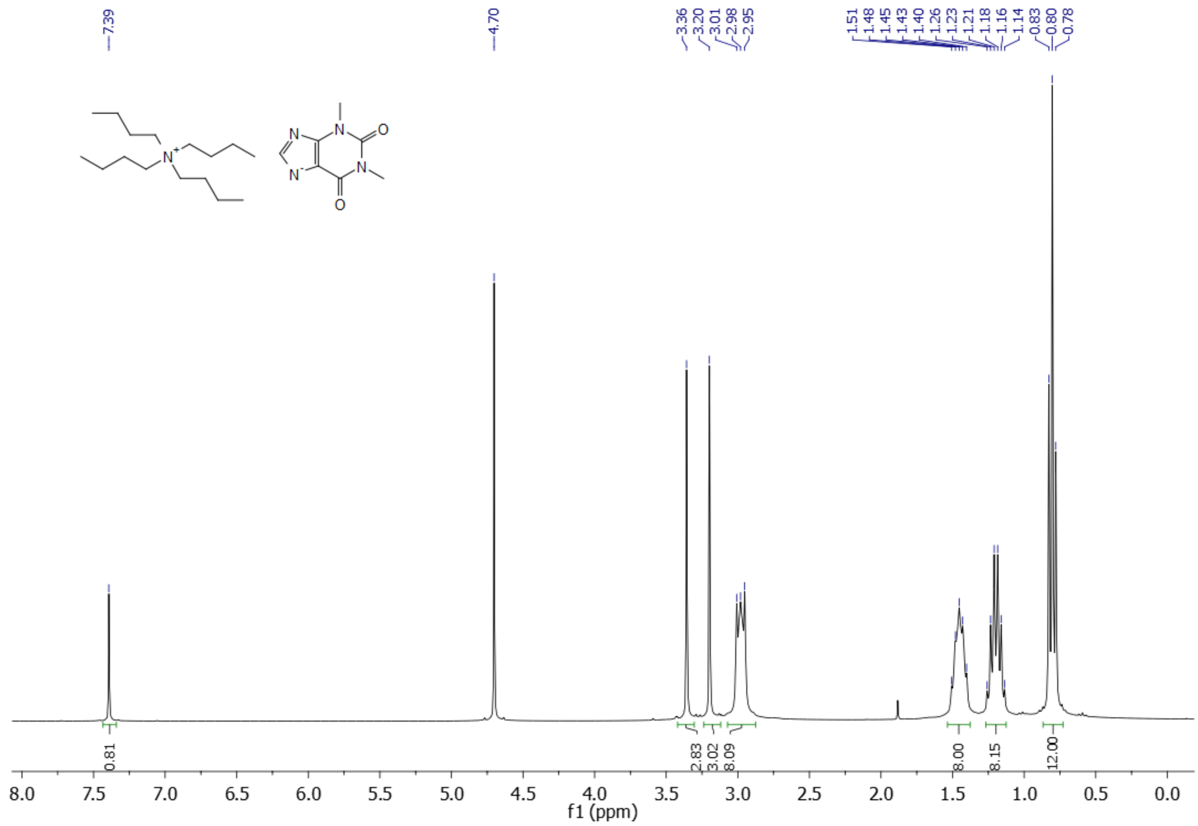


**Spectrum S3.** $^{1}H$ NMR spectra of [$N_{4444}$][Theop] ($D_2O$, 300 MHz).

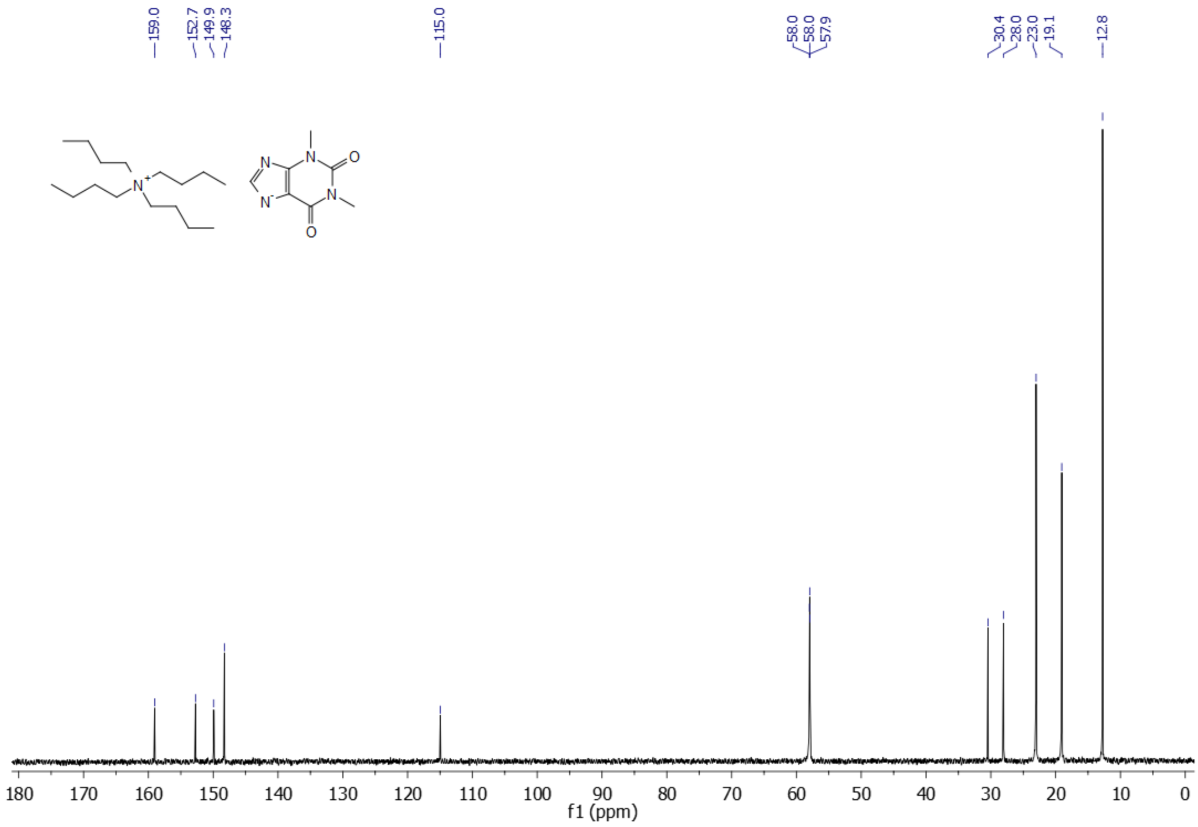


**Spectrum S4.** $^{13}C$ NMR spectra of [$N_{4444}$][Theop] ($D_2O$, 75.47 MHz).

**Tetrabutylammonium xanthinate, [$N_{4444}$][Xan]:**

$^1$H NMR ($D_2O$, 300 MHz, [ppm]): δ 0.76 (t, $J_{HH}$ = 7.3 Hz, 12H, $N^+CH_2CH_2CH_2C\boldsymbol{H}_3$), 1.16 (sext, $J_{HH}$ = 7.3 Hz, 8H, $N^+CH_2CH_2C\boldsymbol{H}_2CH_3$), 1.37 – 1.47 (m, 8H, $N^+CH_2C\boldsymbol{H}_2CH_2CH_3$), 2.93 – 2.98 (m, 8H, $N^+C\boldsymbol{H}_2CH_2CH_2CH_3$), 7.57 (s, 1H, -NC***H***N-). $^{13}$C NMR ($D_2O$, 75.47 MHz, [ppm]): δ 12.7, 19.0, 23.0, 57.9, 113.3, 138.8, 154.3, 159.0, 160.4.

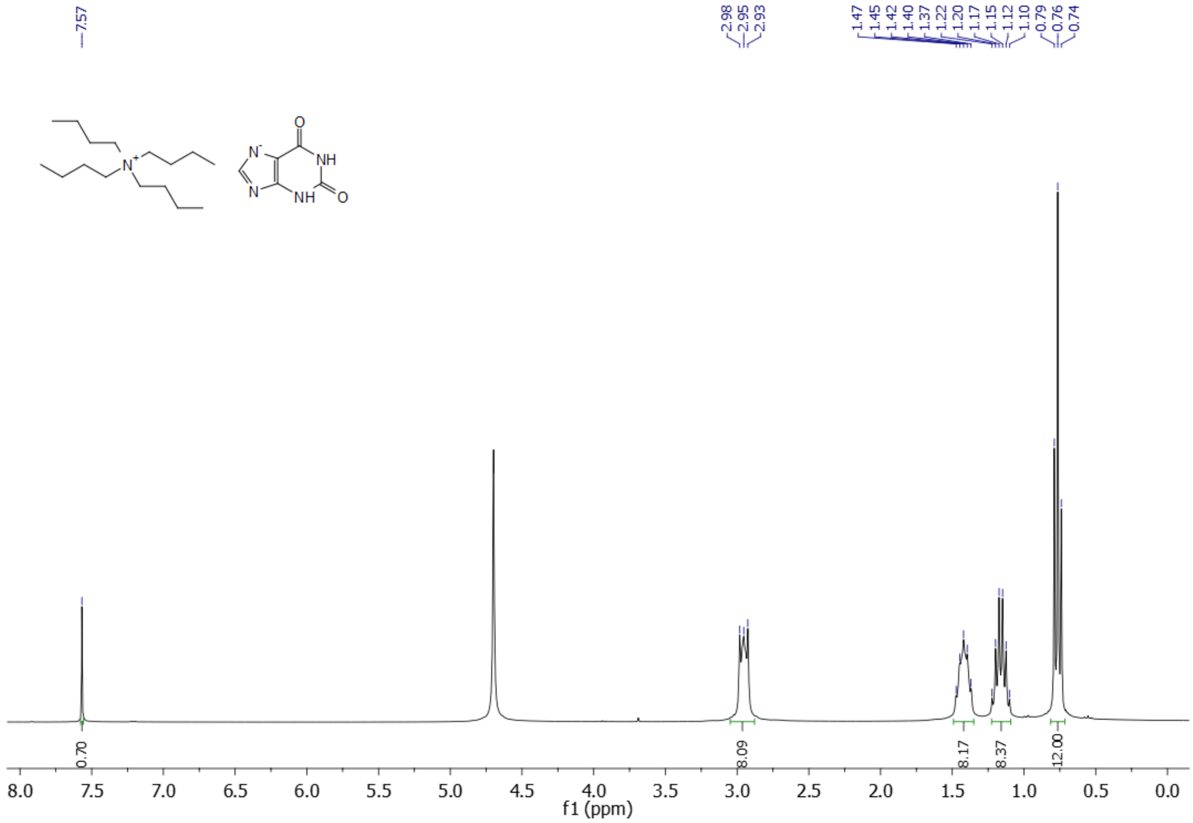


**Spectrum S5.** $^1$H NMR spectra of [$N_{4444}$][Xan] ($D_2O$, 300 MHz).

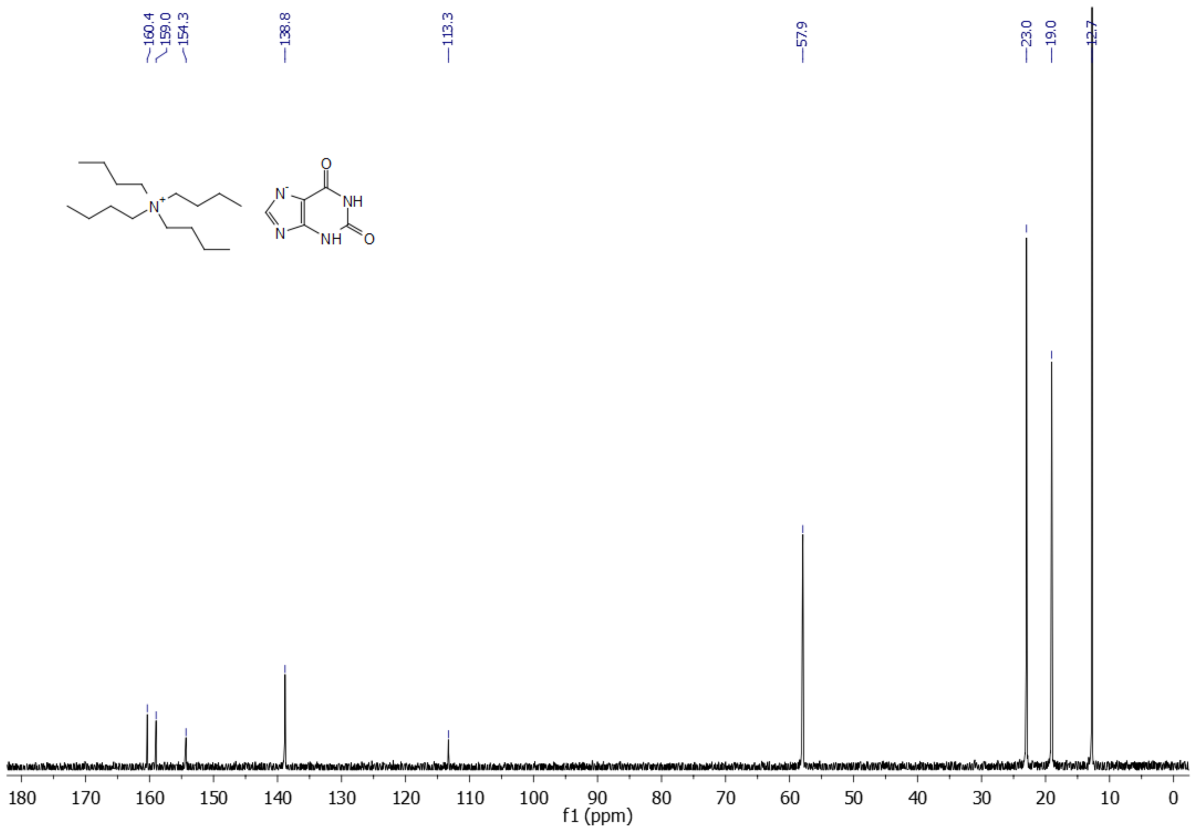


**Spectrum S6.** $^{13}C$ NMR spectra of $[N_{4444}]$[Xan] ($D_2O$, 75.47 MHz).

**Tetrabutylammonium urate, $[N_{4444}]$[Ur]:**

$^1H$ NMR ($D_2O$, 300 MHz, [ppm]): δ 0.79 (t, $J_{HH}$ = 7.3 Hz, 12H, $N^+CH_2CH_2CH_2\boldsymbol{CH_3}$), 1.16 (sext, $J_{HH}$ = 7.2 Hz, 8H, $N^+CH_2CH_2\boldsymbol{CH_2}CH_3$), 1.46 (quint, $J_{HH}$ = 15.4, 8.0 Hz, 8H, $N^+CH_2\boldsymbol{CH_2}CH_2CH_3$), 2.97 – 3.03 (m, 8H, $N^+\boldsymbol{CH_2}CH_2CH_2CH_3$). $^{13}C$ NMR ($D_2O$, 75.47 MHz, [ppm]): δ 12.7, 19.1, 23.0, 58.0, 98.6, 151.1, 154.8, 155.5, 158.8.

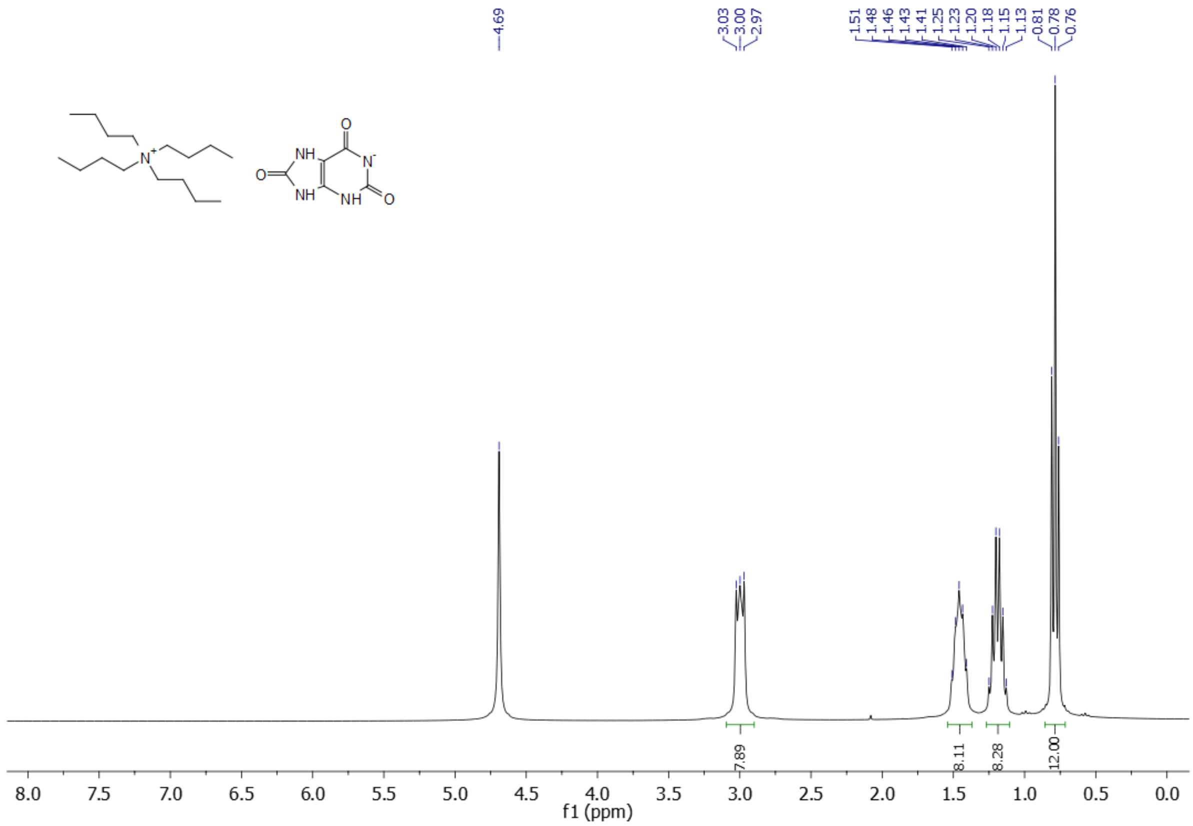


**Spectrum S7.** $^{1}$H NMR spectra of [$N_{4444}$][Ur] ($D_2O$, 300 MHz).

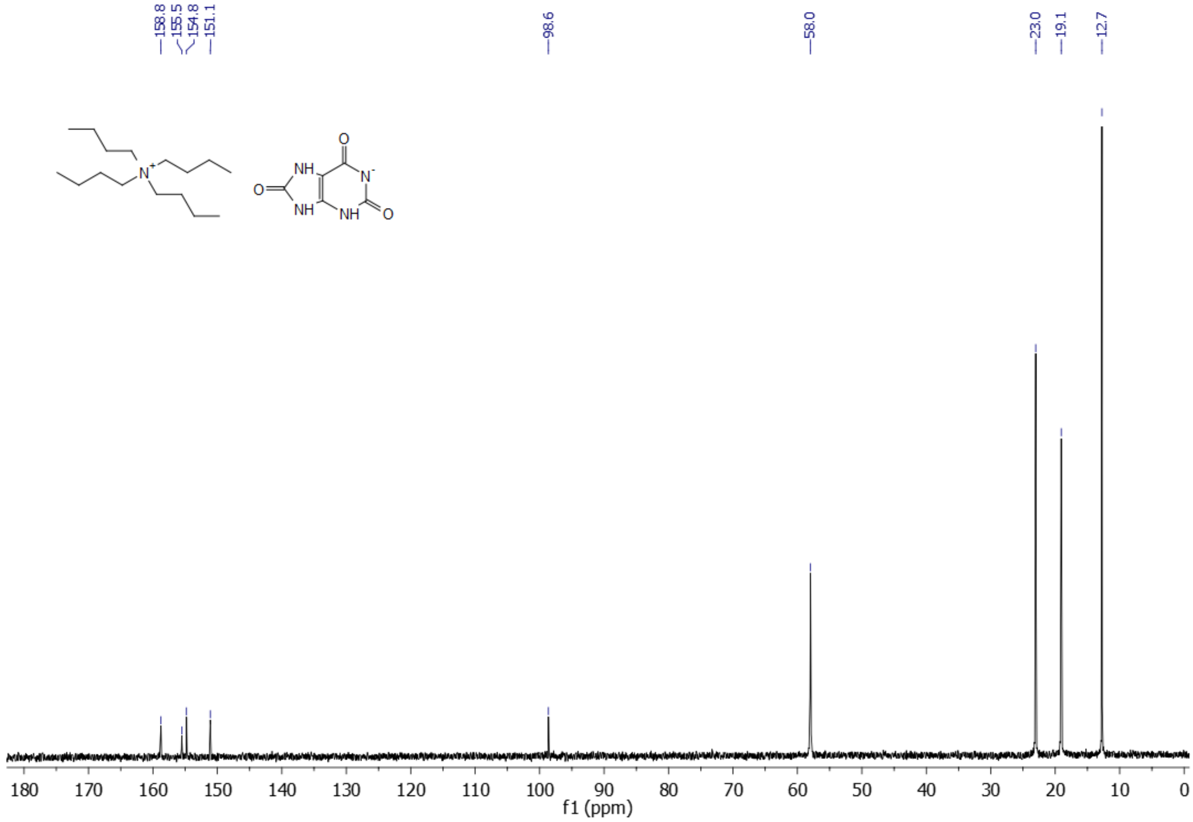


**Spectrum S8.** $^{13}$C NMR spectra of [$N_{4444}$][Ur] ($D_2O$, 75.47 MHz).

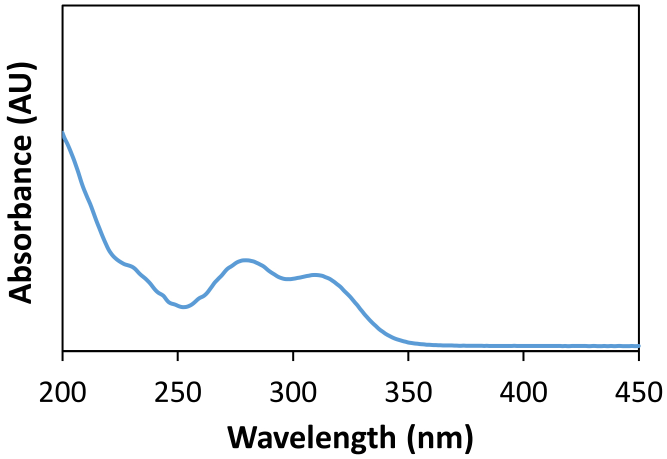


**Figure S1.** Ferulic acid absorbance spectrum in an aqueous solution of $[N_{4444}]$[Theob] at 0.5 mol·L$^{-1}$.

**Table S1.** Molar water-solubility ($S$) of the purine-based ILs, pH of the saturated IL solution at (298 ± 1) K and molar water-solubility of their respective purines ($S_0$).

| IL | $S \pm \sigma$ (mol·L$^{-1}$) | pH saturated | Purine | $S_0$ (mol·L$^{-1}$)[a] |
|---|---|---|---|---|
| $[N_{4444}]$[Theob] | 1.59 ± 0.07 | 12.76 | Theobromine | $1.83 \times 10^{-3}$ [b] |
| $[N_{4444}]$[Theop] | 2.2 ± 0.1 | 10.89 | Theophylline | $4.09 \times 10^{-2}$ [b] |
| $[N_{4444}]$[Xan] | 0.17 ± 0.01 | 10.14 | Xanthine | $4.60 \times 10^{-4}$ [c] |
| $[N_{4444}]$[Ur] | 0.096 ± 0.007 | 8.31 | Uric acid | $3.57 \times 10^{-4}$ [d] |

[a] Data from PubChem [1]. [b] Solubility measured at (298 ± 1) K. [c] Solubility determined at (289 ± 1) K. [d] Solubility determined at (293 ± 1) K.

**Table S2.** Molar solubility ($S$) of sodium theophyllinate in an aqueous solution of $[N_{4444}]$Cl (1.0 mol·L$^{-1}$) and in pure water ($S_0$) and its molar aqueous solubility enhancement ($S/S_0$). The pH of the initial aqueous solution of $[N_{4444}]$Cl and of the final saturated solution are also shown.

| $S \pm \sigma$ (mol·L$^{-1}$) | $S_0 \pm \sigma$ (mol·L$^{-1}$) | $S/S_0$ | pH initial | pH saturated |
|---|---|---|---|---|
| 0.30 ± 0.02 | 0.26 ± 0.01 | 1.13 | 4.23 | 10.55 |

**Table S3.** $EC_{50}$ values ($mg \cdot L^{-1}$) of the purine-based ILs and $[N_{4444}]Cl$, after 96 h of incubation with the microalgae *Raphidocelis subcapitata* and the logarithm function of their anion octanol-water partition coefficient, log ($K_{ow}$). The respective 95% confidence limits are presented in the parenthesis.

| IL | $EC_{50}$ / ($mg \cdot L^{-1}$)<br>(lower limit; upper limit) | log ($K_{ow}$)[a] |
|---|---|---|
| $[N_{4444}]$[Theob] | 2.6<br>(1.8; 3.3) | -3.17 |
| $[N_{4444}]$[Theop] | 3.7<br>(1.4; 5.9) | -2.12 |
| $[N_{4444}]$[Xan] | 0.8<br>(0.7; 1.0) | -1.49 |
| $[N_{4444}]$[Ur] | 2.7<br>(1.6; 3.7) | -2.75 |
| $[N_{4444}]Cl$ | 2.6<br>(2.0; 3.1) | 0.96 |

[a] Data from Marvin 21.14 [2].

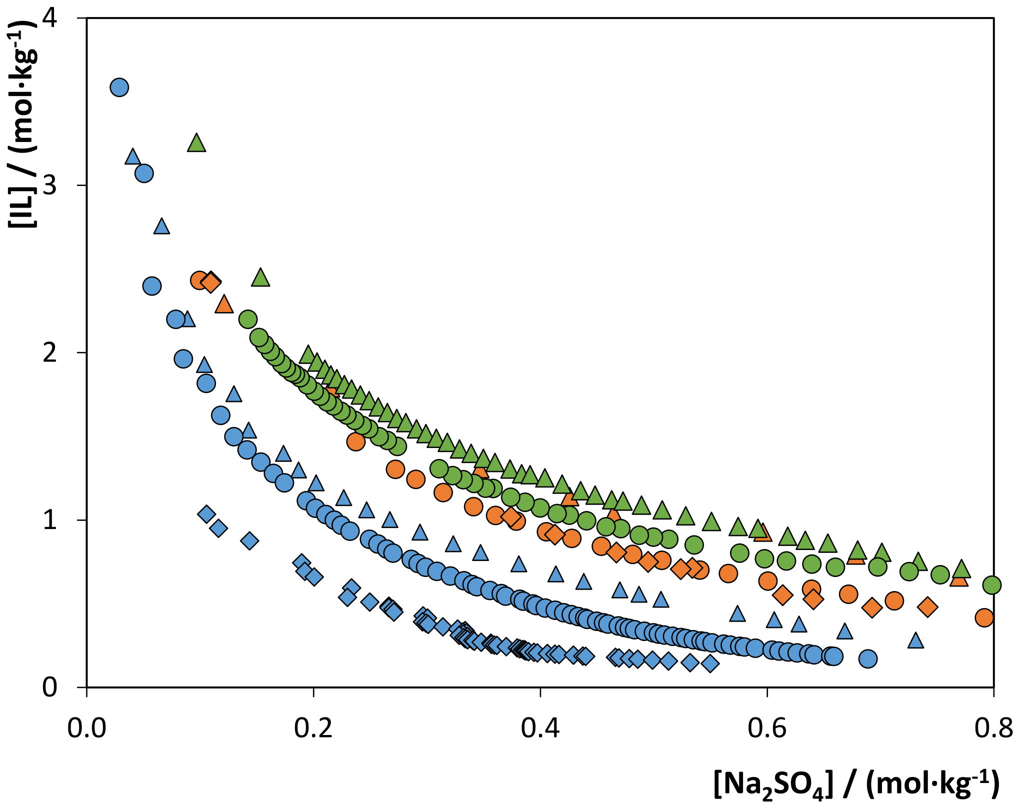


**Figure S2.** Binodal curves of the ternary systems composed of $[N_{4444}]$[Theop] (blue), $[N_{4444}]$[Theob] (orange) or $[N_{4444}]$Cl (green), water and $Na_2SO_4$ at 298 K (Δ), 323 K (○) or 353 K (◇) (± 1 K) and atmospheric pressure (0.1 MPa). $[N_{4444}]$Cl was not tested at (353 ± 1) K.

**Table S4.** Experimental weight fraction of the ternary systems composed of IL, water and $Na_2SO_4$ at (298 ± 1) K and atmospheric pressure (0.1 MPa).

| $[N_{4444}]Cl$ | | | | | |
|---|---|---|---|---|---|
| **IL (wt%)** | **$Na_2SO_4$ (wt%)** | **IL (wt%)** | **$Na_2SO_4$ (wt%)** | **IL (wt%)** | **$Na_2SO_4$ (wt%)** |
| 47.51 | 1.36 | 29.65 | 4.07 | 23.25 | 6.50 |
| 40.54 | 2.13 | 29.27 | 4.19 | 22.76 | 6.72 |
| 35.63 | 2.70 | 28.92 | 4.32 | 22.19 | 6.98 |
| 35.08 | 2.80 | 28.38 | 4.46 | 21.59 | 7.25 |
| 34.56 | 2.89 | 28.00 | 4.59 | 21.08 | 7.54 |
| 34.17 | 2.97 | 27.55 | 4.73 | 20.89 | 7.75 |
| 33.90 | 3.04 | 27.21 | 4.86 | 20.07 | 8.07 |
| 33.46 | 3.13 | 26.62 | 5.03 | 19.64 | 8.25 |
| 33.13 | 3.21 | 26.18 | 5.16 | 19.31 | 8.49 |
| 32.68 | 3.31 | 26.09 | 5.26 | 18.59 | 8.80 |
| 32.27 | 3.41 | 25.83 | 5.42 | 18.32 | 9.06 |
| 31.79 | 3.52 | 25.25 | 5.62 | 17.31 | 9.43 |
| 31.35 | 3.63 | 24.61 | 5.82 | 16.48 | 9.88 |
| 30.88 | 3.74 | 24.21 | 5.99 | 16.32 | 10.39 |
| 30.55 | 3.84 | 23.75 | 6.17 | | |
| **$[N_{4444}]$[Theob]** | | | | | |
| **IL (wt%)** | **$Na_2SO_4$ (wt%)** | **IL (wt%)** | **$Na_2SO_4$ (wt%)** | **IL (wt%)** | **$Na_2SO_4$ (wt%)** |
| 49.16 | 1.69 | 30.46 | 6.19 | 20.45 | 10.44 |
| 43.02 | 2.96 | 28.10 | 7.81 | 18.05 | 11.36 |
| 35.51 | 4.69 | 25.01 | 8.79 | 17.09 | 11.98 |
| 32.45 | 5.70 | 21.76 | 9.85 | 15.88 | 12.68 |
| **$[N_{4444}]$[Theop]** | | | | | |
| **IL (wt%)** | **$Na_2SO_4$ (wt%)** | **IL (wt%)** | **$Na_2SO_4$ (wt%)** | **IL (wt%)** | **$Na_2SO_4$ (wt%)** |
| 57.24 | 0.57 | 32.35 | 3.12 | 19.72 | 6.26 |
| 53.77 | 0.93 | 30.92 | 3.38 | 19.01 | 6.47 |
| 48.17 | 1.24 | 29.70 | 3.66 | 18.15 | 6.71 |
| 44.85 | 1.45 | 28.14 | 4.00 | 15.73 | 7.53 |
| 42.53 | 1.81 | 26.57 | 4.39 | 14.56 | 7.93 |
| 39.33 | 1.99 | 25.35 | 4.70 | 13.77 | 8.19 |
| 37.09 | 2.40 | 23.76 | 5.13 | 12.46 | 8.67 |
| 35.38 | 2.58 | 22.23 | 5.55 | 10.63 | 9.41 |
| 34.02 | 2.79 | 21.06 | 5.86 | | |

**Table S5.** Experimental weight fraction of the ternary systems composed of IL, water and $Na_2SO_4$ at (323 ± 1) K and atmospheric pressure (0.1 MPa).

| $[N_{4444}]Cl$ | | | | | |
|---|---|---|---|---|---|
| **IL (wt%)** | **$Na_2SO_4$ (wt%)** | **IL (wt%)** | **$Na_2SO_4$ (wt%)** | **IL (wt%)** | **$Na_2SO_4$ (wt%)** |
| 37.93 | 1.98 | 30.33 | 3.33 | 20.86 | 6.27 |
| 36.75 | 2.11 | 30.03 | 3.41 | 20.17 | 6.47 |
| 36.28 | 2.18 | 29.41 | 3.53 | 19.98 | 6.63 |
| 35.81 | 2.25 | 29.09 | 3.63 | 19.75 | 6.79 |
| 35.45 | 2.31 | 28.58 | 3.74 | 19.11 | 7.07 |

| | | | | | |
|---|---|---|---|---|---|
| 34.98 | 2.38 | 26.65 | 4.23 | 18.23 | 7.56 |
| 34.66 | 2.44 | 26.01 | 4.38 | 17.60 | 7.82 |
| 34.35 | 2.50 | 25.65 | 4.50 | 17.33 | 8.05 |
| 34.21 | 2.54 | 25.29 | 4.62 | 16.99 | 8.33 |
| 33.95 | 2.60 | 24.87 | 4.76 | 16.68 | 9.01 |
| 33.43 | 2.69 | 24.84 | 4.84 | 16.64 | 8.57 |
| 32.98 | 2.77 | 24.01 | 5.04 | 16.14 | 9.34 |
| 32.60 | 2.84 | 23.50 | 5.20 | 15.73 | 9.66 |
| 32.17 | 2.92 | 22.95 | 5.37 | 14.51 | 10.18 |
| 31.85 | 3.00 | 22.41 | 5.56 | 14.03 | 10.57 |
| 31.45 | 3.08 | 22.23 | 5.70 | 13.43 | 11.07 |
| 31.15 | 3.15 | 21.68 | 5.89 | | |
| 30.71 | 3.24 | 21.07 | 6.11 | | |
| **[$N_{4444}$][Theob]** | | | | | |
| **IL (wt%)** | **$Na_2SO_4$ (wt%)** | **IL (wt%)** | **$Na_2SO_4$ (wt%)** | **IL (wt%)** | **$Na_2SO_4$ (wt%)** |
| 50.64 | 1.40 | 28.17 | 5.44 | 19.84 | 8.32 |
| 38.22 | 3.26 | 27.28 | 5.73 | 18.99 | 8.71 |
| 35.46 | 3.72 | 26.23 | 6.05 | 17.88 | 9.19 |
| 34.38 | 3.96 | 25.08 | 6.40 | 14.94 | 10.11 |
| 32.92 | 4.27 | 24.22 | 6.72 | 13.81 | 10.69 |
| 31.27 | 4.62 | 22.83 | 7.13 | 12.44 | 11.33 |
| 30.21 | 4.87 | 22.27 | 7.44 | | |
| 29.51 | 5.10 | 21.13 | 7.86 | | |
| **[$N_{4444}$][Theop]** | | | | | |
| **IL (wt%)** | **$Na_2SO_4$ (wt%)** | **IL (wt%)** | **$Na_2SO_4$ (wt%)** | **IL (wt%)** | **$Na_2SO_4$ (wt%)** |
| 60.19 | 0.40 | 22.63 | 4.20 | 12.67 | 6.42 |
| 56.43 | 0.71 | 21.91 | 4.35 | 12.36 | 6.53 |
| 50.28 | 0.81 | 21.16 | 4.51 | 12.07 | 6.63 |
| 48.11 | 1.10 | 20.60 | 4.60 | 11.86 | 6.68 |
| 45.30 | 1.19 | 20.22 | 4.66 | 11.63 | 6.75 |
| 43.38 | 1.47 | 19.62 | 4.81 | 11.38 | 6.83 |
| 40.66 | 1.65 | 19.09 | 4.93 | 11.12 | 6.93 |
| 38.71 | 1.81 | 18.71 | 4.98 | 10.93 | 6.98 |
| 37.43 | 1.97 | 18.16 | 5.14 | 10.70 | 7.06 |
| 36.22 | 2.13 | 17.87 | 5.18 | 10.48 | 7.14 |
| 35.04 | 2.28 | 17.37 | 5.29 | 10.32 | 7.18 |
| 34.01 | 2.41 | 17.07 | 5.33 | 10.10 | 7.26 |
| 31.98 | 2.67 | 16.65 | 5.43 | 9.81 | 7.39 |
| 31.11 | 2.78 | 16.24 | 5.54 | 9.62 | 7.46 |
| 30.35 | 2.90 | 15.85 | 5.63 | 9.39 | 7.56 |
| 29.62 | 3.01 | 15.50 | 5.72 | 9.22 | 7.61 |
| 28.99 | 3.09 | 15.16 | 5.80 | 8.97 | 7.73 |
| 28.23 | 3.19 | 14.85 | 5.87 | 8.56 | 7.90 |
| 27.19 | 3.42 | 14.64 | 5.90 | 8.39 | 7.98 |
| 26.51 | 3.52 | 14.29 | 6.00 | 8.18 | 8.07 |
| 25.85 | 3.62 | 14.01 | 6.08 | 7.98 | 8.17 |
| 25.27 | 3.69 | 13.77 | 6.13 | 7.75 | 8.29 |
| 24.36 | 3.90 | 13.39 | 6.24 | 7.61 | 8.35 |
| 23.77 | 3.99 | 13.12 | 6.31 | 7.32 | 8.52 |

| | | | | | |
|---|---|---|---|---|---|
| 23.24 | 4.07 | 12.91 | 6.36 | 7.21 | 8.56 |

**Table S6.** Experimental weight fraction of the ternary systems composed of IL, water and $Na_2SO_4$ at (353 ± 1) K.

| **[$N_{4444}$][Theob]** | | | | | |
|---|---|---|---|---|---|
| **IL (wt%)** | **$Na_2SO_4$ (wt%)** | **IL (wt%)** | **$Na_2SO_4$ (wt%)** | **IL (wt%)** | **$Na_2SO_4$ (wt%)** |
| 50.57 | 1.53 | 22.93 | 6.92 | 11.01 | 11.29 |
| 50.47 | 1.53 | 23.14 | 7.05 | 6.14 | 12.55 |
| 30.07 | 5.04 | 18.83 | 8.02 | 4.60 | 13.40 |
| 27.80 | 5.54 | 18.18 | 8.34 | 1.80 | 15.24 |
| 25.35 | 6.22 | 16.71 | 8.96 | 0.78 | 16.98 |
| 24.03 | 6.57 | 16.81 | 9.53 | | |
| **[$N_{4444}$][Theop]** | | | | | |
| **IL (wt%)** | **$Na_2SO_4$ (wt%)** | **IL (wt%)** | **$Na_2SO_4$ (wt%)** | **IL (wt%)** | **$Na_2SO_4$ (wt%)** |
| 60.19 | 0.40 | 21.91 | 4.35 | 12.07 | 6.63 |
| 56.43 | 0.71 | 21.16 | 4.51 | 11.86 | 6.68 |
| 50.28 | 0.81 | 20.60 | 4.60 | 11.63 | 6.75 |
| 48.11 | 1.10 | 20.22 | 4.66 | 11.38 | 6.83 |
| 45.30 | 1.19 | 19.62 | 4.81 | 11.12 | 6.93 |
| 43.38 | 1.47 | 19.09 | 4.93 | 10.93 | 6.98 |
| 40.66 | 1.65 | 18.71 | 4.98 | 10.70 | 7.06 |
| 38.71 | 1.81 | 18.16 | 5.14 | 10.48 | 7.14 |
| 37.43 | 1.97 | 17.87 | 5.18 | 10.32 | 7.18 |
| 36.22 | 2.13 | 17.37 | 5.29 | 10.10 | 7.26 |
| 35.04 | 2.28 | 17.07 | 5.33 | 9.81 | 7.39 |
| 34.01 | 2.41 | 16.65 | 5.43 | 9.62 | 7.46 |
| 31.98 | 2.67 | 16.24 | 5.54 | 9.39 | 7.56 |
| 31.11 | 2.78 | 15.85 | 5.63 | 9.22 | 7.61 |
| 30.35 | 2.90 | 15.50 | 5.72 | 8.97 | 7.73 |
| 29.62 | 3.01 | 15.16 | 5.80 | 8.56 | 7.90 |
| 28.99 | 3.09 | 14.85 | 5.87 | 8.39 | 7.98 |
| 28.23 | 3.19 | 14.64 | 5.90 | 8.18 | 8.07 |
| 27.19 | 3.42 | 14.29 | 6.00 | 7.98 | 8.17 |
| 26.51 | 3.52 | 14.01 | 6.08 | 7.75 | 8.29 |
| 25.85 | 3.62 | 13.77 | 6.13 | 7.61 | 8.35 |
| 25.27 | 3.69 | 13.39 | 6.24 | 7.32 | 8.52 |
| 24.36 | 3.90 | 13.12 | 6.31 | 7.21 | 8.56 |
| 23.77 | 3.99 | 12.91 | 6.36 | 6.70 | 8.91 |
| 23.24 | 4.07 | 12.67 | 6.42 | | |
| 22.63 | 4.20 | 12.36 | 6.53 | | |

**Table S7.** Experimental weight fraction of the ternary systems composed of IL, water and $K_3C_6H_5O_7$ at (298 ± 1) K.

| **[$N_{4444}$][Theob]** | | | | | |
|---|---|---|---|---|---|
| **IL (wt%)** | **$K_3C_6H_5O_7$ (wt%)** | **IL (wt%)** | **$K_3C_6H_5O_7$ (wt%)** | **IL (wt%)** | **$K_3C_6H_5O_7$ (wt%)** |
| 53.97 | 5.53 | 34.98 | 13.12 | 18.96 | 23.48 |

| | | | | | |
|---|---|---|---|---|---|
| 53.10 | 5.44 | 33.07 | 14.58 | 18.87 | 23.35 |
| 51.23 | 6.66 | 32.67 | 14.40 | 17.63 | 24.44 |
| 49.87 | 6.48 | 32.13 | 14.82 | 17.56 | 24.35 |
| 48.08 | 7.68 | 31.82 | 14.68 | 16.78 | 25.05 |
| 47.39 | 7.57 | 30.32 | 15.87 | 16.67 | 24.89 |
| 46.58 | 8.13 | 30.11 | 15.76 | 15.15 | 26.26 |
| 45.95 | 8.01 | 28.83 | 16.78 | 15.08 | 26.14 |
| 44.56 | 8.98 | 28.52 | 16.60 | 14.03 | 27.10 |
| 43.90 | 8.84 | 25.99 | 18.66 | 13.97 | 26.99 |
| 42.39 | 9.92 | 25.80 | 18.53 | 13.07 | 27.83 |
| 41.72 | 9.76 | 24.42 | 19.68 | 13.02 | 27.73 |
| 39.96 | 11.03 | 24.20 | 19.51 | 12.07 | 28.62 |
| 39.42 | 10.88 | 21.90 | 21.45 | 12.02 | 28.51 |
| 37.74 | 12.12 | 21.80 | 21.35 | 10.90 | 29.58 |
| 37.20 | 11.95 | 20.90 | 22.12 | 10.85 | 29.46 |
| 35.43 | 13.28 | 20.74 | 21.94 | | |
| **[$N_{4444}$][Theop]** | | | | | |
| **IL (wt%)** | **$K_3C_6H_5O_7$ (wt%)** | **IL (wt%)** | **$K_3C_6H_5O_7$ (wt%)** | **IL (wt%)** | **$K_3C_6H_5O_7$ (wt%)** |
| 59.93 | 0.82 | 17.98 | 11.66 | 10.85 | 15.10 |
| 55.87 | 1.77 | 17.72 | 11.80 | 10.75 | 15.16 |
| 49.96 | 2.53 | 17.38 | 11.90 | 10.60 | 15.31 |
| 45.22 | 3.17 | 17.02 | 12.19 | 10.50 | 15.37 |
| 42.63 | 3.77 | 16.68 | 12.13 | 10.37 | 15.48 |
| 39.83 | 4.30 | 16.38 | 12.32 | 10.23 | 15.55 |
| 37.83 | 4.83 | 16.14 | 12.42 | 10.11 | 15.61 |
| 36.10 | 5.20 | 15.70 | 12.67 | 10.10 | 15.69 |
| 35.05 | 5.59 | 15.38 | 12.70 | 10.00 | 15.71 |
| 33.54 | 5.94 | 15.14 | 12.84 | 9.86 | 15.83 |
| 32.60 | 6.33 | 14.92 | 12.97 | 9.78 | 16.05 |
| 31.25 | 6.60 | 14.70 | 13.08 | 9.34 | 16.32 |
| 30.46 | 6.89 | 14.51 | 13.16 | 9.02 | 16.59 |
| 29.67 | 7.26 | 14.31 | 13.25 | 8.79 | 16.83 |
| 28.54 | 7.53 | 14.10 | 13.37 | 8.60 | 17.09 |
| 27.86 | 7.82 | 13.92 | 13.47 | 8.23 | 17.38 |
| 27.20 | 8.10 | 13.73 | 13.58 | 8.07 | 17.86 |
| 26.47 | 8.32 | 13.48 | 13.60 | 7.86 | 18.33 |
| 25.85 | 8.62 | 13.31 | 13.70 | 7.59 | 18.87 |
| 25.04 | 8.78 | 13.08 | 13.93 | 7.27 | 19.28 |
| 24.48 | 9.05 | 12.93 | 14.00 | 6.85 | 19.70 |
| 23.59 | 9.40 | 12.62 | 14.17 | 6.48 | 20.11 |
| 22.68 | 9.75 | 12.48 | 14.25 | 6.28 | 20.66 |
| 22.17 | 9.88 | 12.33 | 14.34 | 6.04 | 21.41 |
| 21.75 | 10.09 | 12.20 | 14.41 | 5.63 | 21.88 |
| 21.32 | 10.30 | 11.96 | 14.55 | 5.43 | 22.48 |
| 20.76 | 10.41 | 11.70 | 14.69 | 5.05 | 23.04 |
| 20.31 | 10.71 | 11.57 | 14.75 | 4.33 | 23.61 |
| 19.69 | 11.06 | 11.45 | 14.82 | 2.16 | 26.10 |
| 19.03 | 11.32 | 11.33 | 14.89 | 1.94 | 29.74 |
| 18.58 | 11.39 | 11.10 | 15.02 | 1.71 | 38.53 |

| | | | | | |
|---|---|---|---|---|---|
| 18.27 | 11.52 | 10.96 | 15.04 | | |

The experimental binodal curves were fitted according to Equation 1 [3]:

$$[\mathrm{CIL}] = A\exp[(B[\mathrm{salt}]^{0.5}) - (C[\mathrm{salt}]^{3})] \quad (1)$$

where [CIL] and [salt] are the fraction percentages of CIL and salt, respectively. *A*, *B*, and *C* correspond to the fitting parameters.

**Table S8.** Merchuk equation parameters (*A*, *B* and *C*) and the respective standard deviations ($\sigma$) for the ternary systems composed of IL, water and $Na_2SO_4$ at 298 K, 323 K and 353 K (± 1 K).

| **IL** | **(T ± 1) K** | ***A* ± *σ*** | ***B* ± *σ*** | **$10^5$ (*C* ± *σ*)** | ***$R^2$*** |
|---|---|---|---|---|---|
| **$[N_{4444}]$[Theob]** | 298 | 78 ± 5 | -0.35 ± 0.04 | 18 ± 5 | 0.9975 |
| | 323 | 89 ± 2 | -0.47 ± 0.01 | 26 ± 3 | 0.9993 |
| | 353 | 87 ± 6 | -0.44 ± 0.04 | 48 ± 9 | 0.9969 |
| **$[N_{4444}]$[Theop]** | 298 | 89 ± 3 | -0.56 ± 0.02 | 46 ± 10 | 0.9982 |
| | 323 | 93 ± 2 | -0.65 ± 0.01 | 124 ± 8 | 0.9982 |
| | 353 | 74 ± 11 | -0.7 ± 0.1 | 355 ± 74 | 0.9805 |
| **$[N_{4444}]$Cl** | 298 | 82 ± 2 | -0.50 ± 0.01 | $5\times10^{-9}$ ± 2 | 0.9963 |
| | 323 | 81 ± 1 | -0.54 ± 0.01 | $5\times10^{-8}$ ± 2 | 0.9993 |

**Table S9.** Merchuk equation parameters (*A*, *B* and *C*) and the respective standard deviations ($\sigma$) for the ternary systems composed of IL, water and $K_3C_5H_6O_7$ at (298 ± 1) K.

| **IL** | **(T ± 1) K** | ***A* ± *σ*** | ***B* ± *σ*** | **$10^5$ (*C* ± *σ*)** | ***$R^2$*** |
|---|---|---|---|---|---|
| $[N_{4444}]$[Theob] | 298 | 105 ± 3 | -0.285 ± 0.001 | 2.7 ± 0.2 | 0.9990 |
| $[N_{4444}]$[Theop] | 298 | 94 ± 4 | -0.42 ± 0.02 | 14 ± 1 | 0.9918 |

**Table S10.** Experimental TLs and TLLs of the ternary systems composed of ILs + $Na_2SO_4$ at 298 K or 323 K (± 1 K) and atmospheric pressure (0.1 MPa). No TLs were determined at (353 ± 1) K.

| | | Mixture point (wt %) | | Top phase (wt %) | | | Bottom phase (wt %) | | | |
|---|---|---|---|---|---|---|---|---|---|---|
| **IL** | **(T ± 1) K** | **[IL]** | **[salt]** | **[IL]** | **[salt]** | **pH** | **[IL]** | **[salt]** | **pH** | **TLL** |
| $[N_{4444}]$[Theob] | 298 | 24.9 | 10.1 | 35.0 | 4.9 | 11.46 | 2.8 | 21.3 | 11.07 | 36.1 |
| | | 25.0 | 12.0 | 41.3 | 3.2 | 11.47 | 0.9 | 25.0 | 10.98 | 45.9 |
| | 323 | 25.0 | 10.0 | 36.0 | 3.6 | 11.43 | 0.2 | 24.5 | 10.93 | 41.5 |
| | | 25.0 | 12.0 | 41.7 | 2.6 | 11.49 | 0.1 | 26.0 | 11.02 | 47.7 |
| $[N_{4444}]$[Theop] | 298 | 25.0 | 5.6 | 39.1 | 2.1 | 9.86 | 13.3 | 8.4 | 9.25 | 26.6 |
| | | 24.7 | 8.1 | 47.9 | 1.2 | 9.81 | 2.3 | 14.8 | 9.32 | 47.6 |
| | 323 | 25.0 | 5.5 | 49.2 | 1.0 | 9.82 | 4.8 | 9.3 | 9.21 | 45.2 |
| | | 25.1 | 8.0 | 57.9 | 0.5 | 9.79 | 0.4 | 13.7 | 9.26 | 59.1 |
| $[N_{4444}]$Cl | 298 | 25.0 | 10.0 | 33.4 | 3.2 | 5.00 | 6.8 | 24.7 | 4.88 | 34.2 |

**Table S11.** Experimental TLs and TLLs of the ternary systems composed of ILs + $K_3C_5H_6O_7$ at (298 ± 1) K and atmospheric pressure (0.1 MPa).

| | | Mixture point (wt%) | | Top phase (wt%) | | | Bottom phase (wt%) | | | |
|---|---|---|---|---|---|---|---|---|---|---|
| **IL** | **(T ± 1) K** | **[IL]** | **[salt]** | **[IL]** | **[salt]** | **pH** | **[IL]** | **[salt]** | **pH** | **TLL** |
| $[N_{4444}]$[Theob] | 298 | 25.0 | 23.6 | 46.7 | 7.9 | 12.50 | 3.5 | 39.1 | 11.95 | 53.2 |
| | | 25.0 | 28.5 | 59.4 | 4.0 | 12.70 | 1.2 | 45.4 | 12.02 | 71.4 |
| $[N_{4444}]$[Theop] | 298 | 25.1 | 23.7 | 69.7 | 0.4 | 11.03 | 0.1 | 36.8 | 10.44 | 78.6 |
| | | 24.9 | 14.1 | 56.2 | 1.5 | 11.20 | 1.6 | 23.5 | 10.65 | 58.9 |

Ferulic acid was used to perform a simplistic study of its partition in different ternary mixtures. To perform this study, the following mixture points were used: $[N_{4444}]$[Theob]/$[N_{4444}]$Cl–$Na_2SO_4$ (25-10) wt%, and $[N_{4444}]$[Theob]/$[N_{4444}]$[Theop]/$[N_{4444}]$Cl–$K_3C_5H_6O_7$ (25-24) wt% at (298 ± 1) K. The water content of each mixture point was substituted by an aqueous solution of ferulic acid with a concentration of 0.6 $g{\cdot}L^{-1}$. The mixture points were centrifuged for 20 min at 3500 rpm and the phases of the mixture points were separated and individually weighed (± $10^{-4}$ g). The amount of ferulic acid in each phase was determined by UV-Vis spectroscopy. All the assays were performed in duplicates. The extraction efficiency (*EE*) was determined as follows:

$$EE\ \% = \frac{m_{Top}}{m_{total}} \times 100$$

where $m_{Top}$ is the amount of ferulic acid in the top phase, and $m_{total}$ is the total amount of ferulic acid in the ternary mixture, respectively.

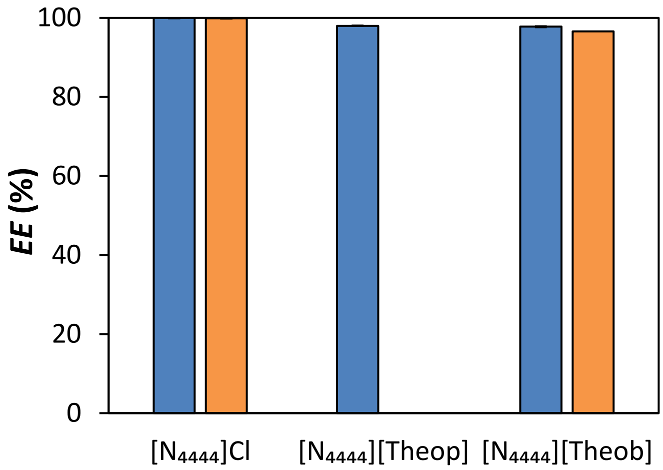


**Figure S3.** Extraction efficiency (*EE* %) of each ternary mixture at (289 ± 1) K and atmospheric pressure (0.1 MPa). Blue bars represent data from systems containing the salting-out agent $K_3C_5H_6O_7$ and orange bars represent ternary mixtures with $Na_2SO_4$ as the salting-out agent.

**Table S12.** Molar solubility (*S*) of ferulic acid in IL solutions with different concentrations.

| ILs | [IL] / (mol·L$^{-1}$) | ($S$ ± $\sigma$) / (mol·L$^{-1}$) |
|---|---|---|
| [$N_{4444}$][Theob] | 0.05 | 0.0144 ± 0.0006 |
| | 0.1 | 0.0247 ± 0.0006 |
| | 0.25 | 0.12 ± 0.02 |
| | 0.5 | 0.23 ± 0.01 |
| [$N_{4444}$][Theop] | 0.05 | 0.0213 ± 0.0002 |
| | 0.1 | 0.063 ± 0.003 |
| | 0.25 | 0.237 ± 0.005 |
| | 0.5 | 0.46 ± 0.04 |
| | 1.0 | 0.60 ± 0.01 |
| | 1.5 | 0.38 ± 0.02 |
| [$N_{4444}$]Cl | 0.05 | 0.0040 ± 0.0001 |
| | 0.1 | 0.0047 ± 0.0001 |
| | 0.25 | 0.034 ± 0.001 |
| | 0.5 | 0.158 ± 0.003 |
| | 1.0 | 0.415 ± 0.009 |
| | 1.5 | 0.24 ± 0.01 |

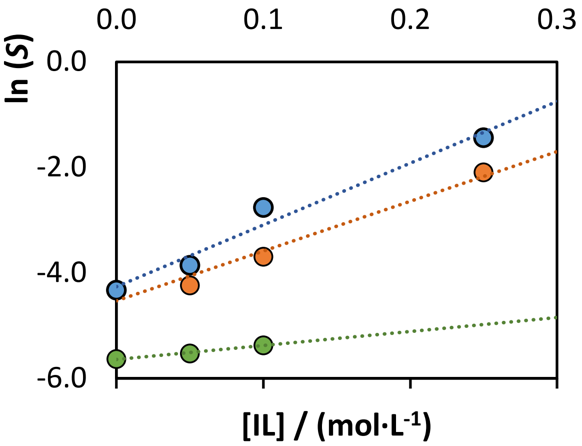


**Figure S4.** Fitted regression to the diluted region solubility data for the determination of the Setschenow constant. Different ILs are represented in different colors: $[N_{4444}]$[Theop] (blue), $[N_{4444}]$[Theob] (orange) and $[N_{4444}]Cl$ (green).

**Table S13.** Setchenow constant for each IL, given by the slope of the fitted regression to the diluted region solubility data, and the respective coefficient of determination ($R^2$).

| IL | Setschenow constant ($L{\cdot}mol^{-1}$) | $R^2$ |
|---|---|---|
| **$[N_{4444}]$[Theob]** | 9.440 | 0.9714 |
| **$[N_{4444}]$[Theop]** | 11.710 | 0.9681 |
| **$[N_{4444}]Cl$** | 2.657 | 0.9900 |

**Table S14.** Measured pH of the IL solutions before the addition of ferulic acid and after obtaining a saturated solution.

| IL | [IL] / (mol·L$^{-1}$) | Initial solution pH | Saturated solution pH |
|---|---|---|---|
| $[N_{4444}][Theob]$ | 0.05 | 10.85 | 5.36 |
| | 0.1 | 11.56 | 5.54 |
| | 0.25 | 11.80 | 8.51 |
| | 0.5 | 11.98 | 9.62 |
| $[N_{4444}][Theop]$ | 0.05 | 8.91 | 5.30 |
| | 0.1 | 10.52 | 6.44 |
| | 0.25 | 10.81 | 6.56 |
| | 0.5 | 10.78 | 7.65 |
| | 1.0 | 11.06 | 8.63 |
| | 1.5 | 10.68 | 9.95 |
| $[N_{4444}]Cl$ | 0.05 | 5.71 | 3.29 |
| | 0.1 | 6.31 | 2.89 |
| | 0.25 | 5.62 | 2.63 |
| | 0.5 | 4.26 | 2.36 |
| | 1.0 | 4.04 | 2.10 |
| | 1.5 | 4.20 | 2.26 |

**Table S15.** Ferulic acid water-solubility at different pH values.

| Initial solution pH | Saturated solution pH | (Solubility ± σ) × 10$^2$ (mol·L$^{-1}$) |
|---|---|---|
| 2.46 | 2.45 | 0.355 ± 0.006 |
| 7.04 | 3.60 | 0.39 ± 0.02 |
| 11.63 | 4.70 | 0.94 ± 0.02 |
| 11.78 | 5.05 | 1.32 ± 0.04 |
| 12.59 | 5.78 | 7.3 ± 0.6 |
| 13.01 | 6.11 | 38 ± 1 |
| 13.16 | 7.93 | 35 ± 2 |
| 13.01 | 8.57 | 33 ± 1 |
| 13.16 | 9.16 | 31 ± 2 |

**References**


1. PubChem Available online: https://pubchem.ncbi.nlm.nih.gov/ (accessed on Mar 8, 2021).
2. MarvinSketch 21.14, 2021, ChemAxon Available online: http://www.chemaxon.com (accessed on Apr 5, 2021).
3. Merchuk, J.C.; Andrews, B.A.; Asenjo, J.A. Aqueous two-phase systems for protein separation studies on phase inversion. *J. Chromatogr. B Biomed. Appl.* **1998**, *711*, 285–293, doi:10.1016/S0378-4347(97)00594-X.
